# Intracellular Retention of ABL Kinase Inhibitors Determines Commitment to Apoptosis in CML Cells

**DOI:** 10.1371/journal.pone.0040853

**Published:** 2012-07-16

**Authors:** Daniel B. Lipka, Marie-Christine Wagner, Marek Dziadosz, Tina Schnöder, Florian Heidel, Mirle Schemionek, Junia V. Melo, Thomas Kindler, Carsten Müller-Tidow, Steffen Koschmieder, Thomas Fischer

**Affiliations:** 1 Department of Hematology and Oncology, University Medical Center, Otto-von-Guericke-University, Magdeburg, Germany; 2 Division of Epigenomics and Cancer Risk Factors, German Cancer Research Center, Heidelberg, Germany; 3 Institute of Forensic Medicine, University Medical Center, Otto-von-Guericke-University, Magdeburg, Germany; 4 Department of Medicine A (Hematology, Oncology and Pneumology), University of Münster, Münster, Germany; 5 Department of Oncology, Hematology and Stem Cell Transplantation, University Medical Center, Rheinisch-Westfaelische Technische Hochschule, Aachen, Germany; 6 Department of Haematology, Centre for Cancer Biology, University of Adelaide, Adelaide, Australia; 7 Department of Haematology, Imperial College London, London, United Kingdom; 8 Third Department of Medicine, University Medical Center, Johannes Gutenberg-University, Mainz, Germany; Mayo Clinic College of Medicine, United States of America

## Abstract

Clinical development of imatinib in CML established continuous target inhibition as a paradigm for successful tyrosine kinase inhibitor (TKI) therapy. However, recent reports suggested that transient potent target inhibition of BCR-ABL by high-dose TKI (HD-TKI) pulse-exposure is sufficient to irreversibly commit cells to apoptosis. Here, we report a novel mechanism of prolonged intracellular TKI activity upon HD-TKI pulse-exposure (imatinib, dasatinib) in BCR-ABL-positive cells. Comprehensive mechanistic exploration revealed dramatic intracellular accumulation of TKIs which closely correlated with induction of apoptosis. Cells were rescued from apoptosis upon HD-TKI pulse either by repetitive drug wash-out or by overexpression of ABC-family drug transporters. Inhibition of ABCB1 restored sensitivity to HD-TKI pulse-exposure. Thus, our data provide evidence that intracellular drug retention crucially determines biological activity of imatinib and dasatinib. These studies may refine our current thinking on critical requirements of TKI dose and duration of target inhibition for biological activity of TKIs.

## Introduction

Chronic myeloid leukemia (CML) is characterized by the constitutively activated tyrosine kinase BCR-ABL. Treatment of CML with the small molecule tyrosine kinase inhibitor (TKI) imatinib stands as a paradigm for clinical efficacy of targeted small molecule therapy in malignant disease. Imatinib inhibits BCR-ABL tyrosine kinase activity and has been shown to effectively target the malignant clone *in vitro* and *in vivo*, resulting in a high percentage of long-term remissions in CML patients. [1] Beyond CML, TKIs are currently either approved or evaluated in numerous other hematologic and solid neoplasms and may become cornerstones of novel treatment strategies in the near future. [2]–[4].

Preclinical and clinical data derived from studies using imatinib and other compounds suggested that candidates for clinical development should exhibit a sufficiently long plasma half-life to facilitate persistent target inhibition: continuous exposure to imatinib concentrations ≥1 µM for at least 20 h is necessary to induce apoptosis in BCR-ABL transformed cells *in vitro*, and clinical trials demonstrated a close relationship between imatinib serum trough-levels and clinical response.[5]–[7] Finally, the extent of BCR-ABL inhibition, as determined by the level of CRKL dephosphorylation, correlated with clinical activity. [8] Therefore, it has been widely accepted that continuous and complete target inhibition is a prerequisite for clinical efficacy of TKI treatment.

Recently, this paradigm has been challenged by data obtained in a clinical trial using the second generation BCR-ABL inhibitor dasatinib. [9] Dasatinib (serum half-life: 3–5 h) demonstrated similar clinical activity but less side effects for once daily dosing with 100 mg as compared to twice daily dosing with 70 mg. [10] Interestingly, the once daily dosing schedule apparently resulted in transient inhibition of BCR-ABL kinase activity only, as re-phosphorylation of the BCR-ABL downstream adaptor protein CRKL was observed 8 h post dasatinib-dosing. [10], [11] In addition, *in vitro* and *ex vivo* studies suggested that high-dose (HD) pulse-exposure to TKI irreversibly commits BCR-ABL positive cells to apoptosis. This effect was evident upon pulse treatment for only 20 min –4 h.[12]–[14] It was proposed that depth, rather than duration of kinase inhibition, is the critical determinant for TKI efficacy. [12], [13] However, the molecular mechanism for apoptosis induction after HD-TKI pulse-exposure has remained elusive.

In our present work, we demonstrate that dramatic intracellular drug retention mediates apoptotic cell death upon HD-TKI pulse-exposure. In line with this, over-expression of ABC transporters prevented cell death upon HD-TKI pulse-exposure. These findings will be useful to rethink our current framework of pharmacokinetic requirements of TKIs for CML and other diseases. In addition, these studies refine the molecular concept of TKI-induced apoptosis.

## Materials and Methods

### Ethics Statement

Patient blood samples were drawn after written informed consent was obtained. Experimentation on human material was performed according to the premises of the Helsinki declaration and was approved by the local ethics committee (University Medical Center, Otto-von-Guericke University Magdeburg, Germany).

**Figure 1:**
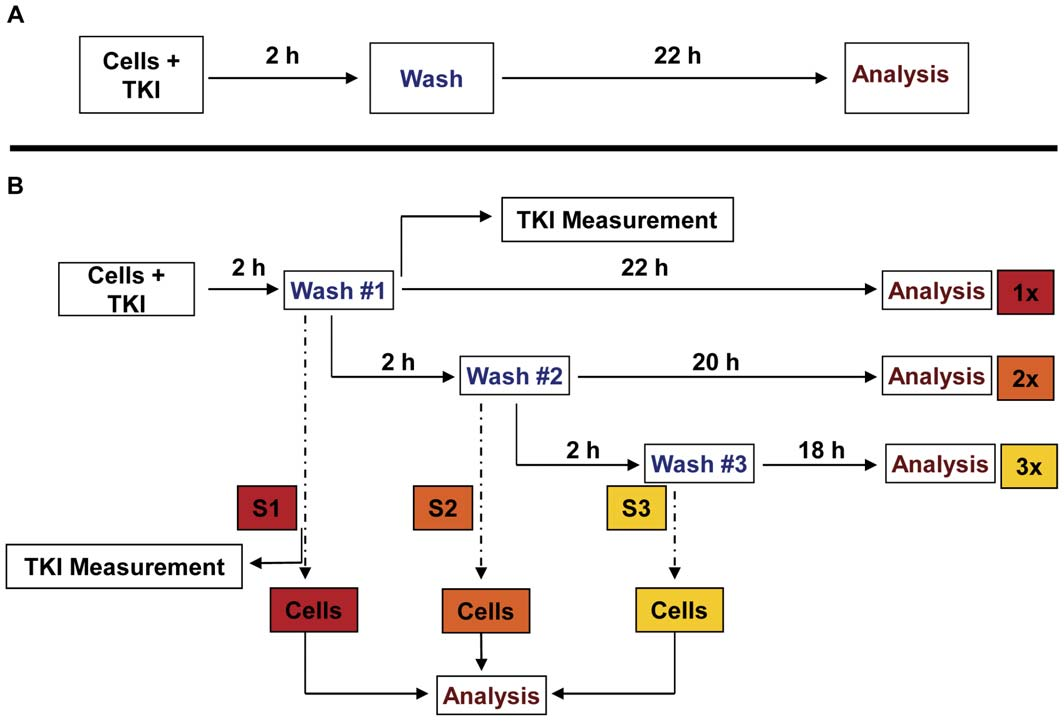
Experimental set-up for analysis of induction of apoptosis upon HD-TKI exposure. (**A**) Cells were seeded at a density of 5×10^4^ cells/ml in a total volume of 2 ml in RPMI 1640 supplemented with 10% FCS. Cells were treated for 2 h with TKI as indicated. Then, cells were washed twice with 2 ml PBS at room temperature and replated in fresh cell culture media (2 ml final volume). Cells exposed to 0.35% DMSO served as controls. 24 h after start of TKI exposure percentage of cells in subG1 phase was measured by flow cytometry after propidium iodide staining. (**B**) To analyze for residual TKI activity upon HD-TKI pulse exposure, a second and third drug wash-out procedure (each consisting of 2×2 ml PBS washing) was performed: Cells were treated with TKI for 2 h. Cells initially pulse-exposed to HD-TKI were washed twice with 2 ml PBS at room temperature and replated in 2 ml fresh media (density: 5×10^4^ cells/ml) as described in (a) (“*1x”*). To test for residual TKI-activity, the cell culture supernatant was transferred to previously untreated cells *(“S1”)*, which were subsequently incubated for 24 h. Two hours after replating, a second drug wash-out was performed (2×2 ml PBS). Cells were again replated in 2 ml fresh media (“*2x”*). Again supernatants were transferred to previously untreated cells (“*S2”*). This procedure was repeated for a third time (“*3x”, “S3”*).

### Cell-lines and Patient Samples

Hematopoietic Ba/F3 cells (DSMZ, Braunschweig, Germany) either parental or stably expressing p210-BCR-ABL (Ba/F3-BCR-ABL) [15] were used. K562 cells were obtained from DSMZ (Braunschweig, Germany). K562-Dox cells (referred herein as K562-ABCG1 cells) were kindly provided by J. Melo (Department of Haematology, Centre for Cancer Biology, University of Adelaide, Australia). [16] K562-ABCG2 cells were kindly provided by Sheng Zhou (Division of Experimental Hematology, Department of Hematology, St. Jude Childreńs Research Hospital, Memphis, USA). [17] Cells were cultured in RPMI1640 containing 4 mM L-glutamine and 10% FCS at 37°C in humidified air containing 5% CO_2_. Media for parental Ba/F3 cells was supplemented with 10% WEHI-conditioned media. Primary CML samples (MNC) as well as CD34^+^ cells from normal controls were isolated and stored in liquid nitrogen. Upon thawing, cells were cultured in RPMI1640 supplemented with 20% FCS, 50 U/ml penicillin, and 50 µg/ml streptomycin.

### Reagents

Cells were treated either with dasatinib (Selleck Chemicals LLC, Houston, TX, USA) or imatinib (kindly provided by Novartis, Basel, Switzerland). A 10 mM stock solution was prepared in DMSO and stored at −20°C. Stocks were further diluted in cell culture medium. The final DMSO concentration was ≤0.35%, depending on the inhibitor concentration used. DMSO exposed cells were used as controls in all experiments. For some experiments 10 µM of the ABCB1 inhibitor PSC833 (Tocris, Missouri, USA) were used.

**Figure 2:**
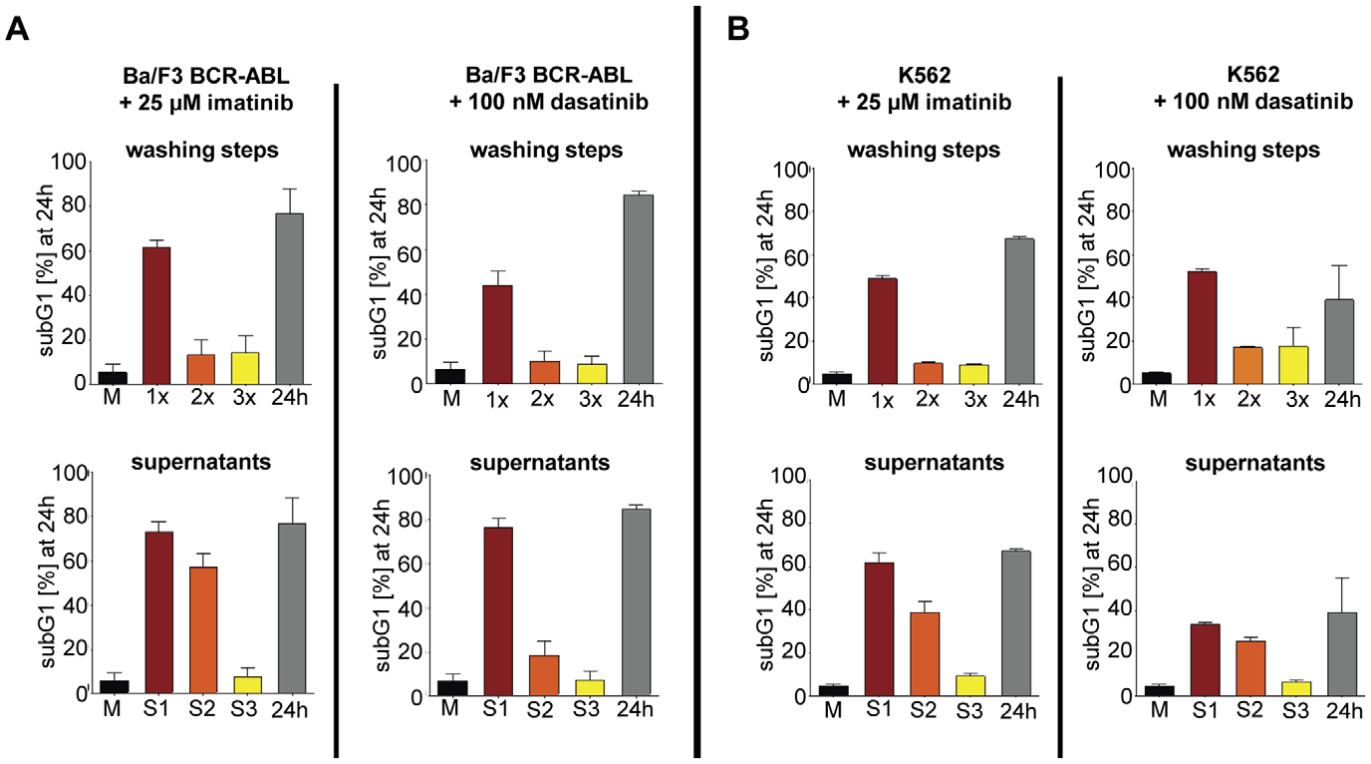
Repetitive washing prevents apoptosis after high-dose TKI pulse-exposure. To analyze for residual TKI activity upon HD-TKI pulse-exposure, we employed repetitive wash-out procedures and exposure of previously untreated cells to cell culture supernatants. Therefore, 5×10^4^ cells/ml were seeded in a total volume of 2 ml in RPMI 1640 supplemented with 10% FCS and treated with TKI as indicated for 2 hours. Cells exposed to 0.35% DMSO served as controls (“M”). Then, cells were washed and replated as described in **Figure 1B** (“1x”, “2x”, “3x”; upper panel). To test for residual TKI-activity, the cell culture supernatants were transferred to previously untreated cells (“S1”, “S2”, “S3”; lower panel). For further details of the experimental procedure please refer to **Figure 1B**. Percentage of cells in subG1 phase was measured by flow cytometry after propidium iodide staining 24 hours after start of TKI treatment and is depicted for Ba/F3-BCR-ABL cells (**A**) and K562 cells (**B**), respectively. Experiments were performed in triplicate. Data are presented as mean percentage of cells in subG1 phase + SEM.

For Western blotting the following antibodies were used: anti-pSTAT5 (pY694) (Millipore, Billerica, MA, USA), anti-STAT5, anti-CRKL, horseradish peroxidase-linked goat anti-mouse immunoglobulin (Santa Cruz, Heidelberg, Germany), anti-pABL (pY412, pY177), anti-ABL, anti-pCRKL (pY207), total caspase3, cleaved caspase3, horseradish peroxidase-linked goat anti-rabbit (Cell Signaling Technology, Danvers, MA, USA), anti-GAPDH (Meridian Lifescience, Saco, ME, USA).

**Figure 3:**
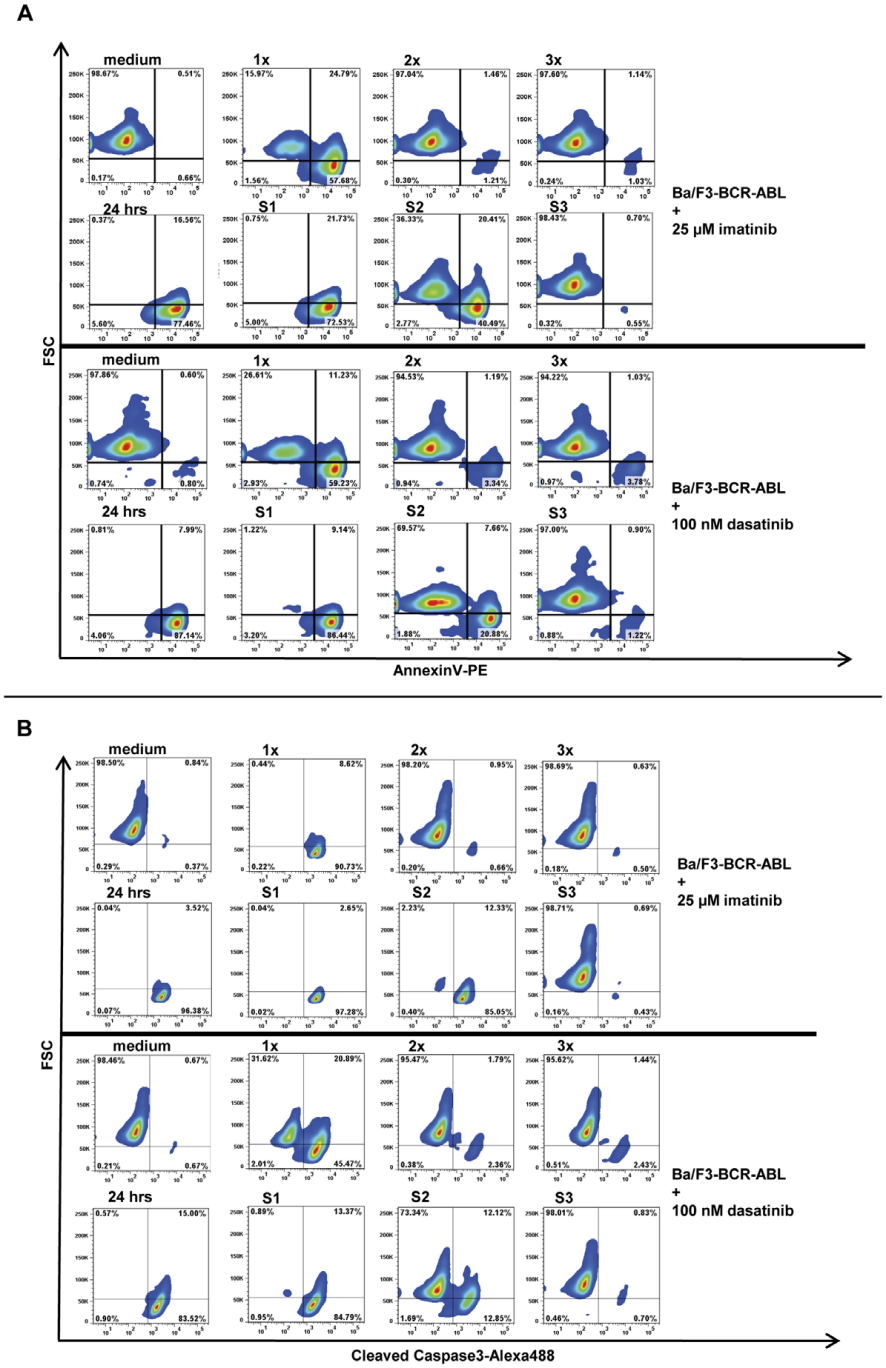
AnnexinV- and Cleaved Caspase3-staining after high-dose TKI pulse-exposure. To confirm results obtained with propidium iodide staining, AnnexinV-PE staining (**A**) and Cleaved Caspase3-Alexa488 staining (**B**) of Ba/F3-BCR-ABL cells was performed at 24 hours after HD-TKI pulse-exposure using either imatinib or dasatinib as indicated followed by repetitive wash-out as described in **Figure 2**. Experiments were performed in triplicate and one representative experiment is shown.

### Experimental Design, TKI Treatment and Drug Wash-out Procedures

If not otherwise stated, cells were seeded at a density of 5×10^4^ cells/ml in a total volume of 2 ml in cell culture media and treated for 2 h with the respective TKI. Following treatment, cells were washed twice with 2 ml PBS at room temperature and subsequently re-seeded in 2 ml cell culture media. The washing procedure was repeated twice, after 2 and 4 h, respectively. The detailed experimental setup is described in **Figure 1**.

**Figure 4:**
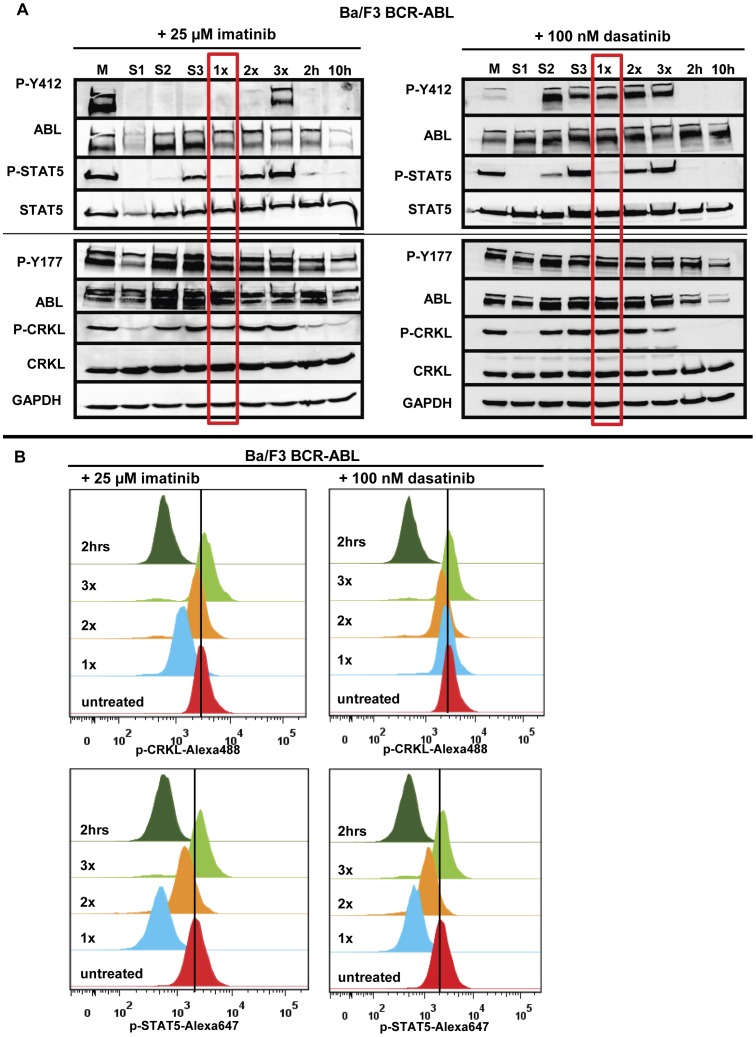
Recovery kinetics of intracellular signaling pathways upon HD-TKI pulse-exposure. (**A**) Ba/F3-BCR-ABL cells were treated either with 25 µM imatinib or with 100 nM dasatinib for 2 hours followed by drug wash-out and consecutive replating as described in **Figure 1B**. Untreated cells served as positive controls for phosphorylation signals (“M”). Cells treated continuously for 2 hours or 10 hours (2 h and 10 h) served as positive controls for dephosphorylation of intracellular signalling pathways. “M”: medium; “1x”, “2x”, “3x”: Cells were treated with HD-TKI for 2 h and drug wash-out was performed 1–3x in 2 h intervals, respectively. Lysates were prepared 2 h after drug wash-out, allowing for recovery of phosphorylation signals in case of absent TKI activity. “S1”, “S2”, “S3”: Lysates were prepared upon incubation of previously untreated cells for 2 h with the respective cell culture supernatants obtained during each washing procedure. (**B**) FACS analysis of Ba/F3-BCR-ABL cells after HD-TKI exposure followed by repetitive drug wash-out (“1x”, “2x”, “3x”) using phosphorylation-specific antibodies against CRKL (upper panel) and STAT5 (lower panel). Cells were treated with respective TKI for 2 h as indicated and washed as explained in **Figure 1B**. Two hours after each washing step cells were fixed and permeabilized. Antibody staining was performed in parallel with p-CRKL-Alexa488 and p-STAT5-Alexa647 antibody. Untreated cells served as a control for phosphorylation signals. All experiments were performed at least in triplicate and one representative experiment is shown.

### Cell Lysate Preparation and Western Blotting

2×10^6^ cells were incubated as described above. Cells were then resuspended in 100 µl lysis buffer (20 mM Tris-HCl; pH 7.0, 150 mM NaCl, 10% (v/v) glycerol, 1% Triton X-100, 1.5 mM EDTA, 1 mM phenylmethylsulfonyl fluoride, 0.2 mM sodium orthovanadate, 10 mM NaF, 0.4x Complete [Roche Diagnostics, Mannheim, Germany], 1x PhosStop [Roche Diagnostics, Mannheim, Germany]) and incubated for 30 min at 4°C. Lysates were then cleared by centrifugation and subsequently subjected to SDS-polyacrylamide gel electrophoresis (PAGE) and blotted onto nitrocellulose membranes (ECL membrane, Amersham, Freiburg, Germany) using an SDS electroblotting system (BioRad, Munich, Germany). Membranes were blocked with PBS/0.5% Tween/5% nonfat dry-milk for 1 h, incubated with primary antibody overnight at 4°C in blocking solution and washed twice in PBS/0.5% Tween. The membranes were then incubated for 1 h with an appropriate secondary antibody and specific proteins were visualized using the ECL detection system (Amersham, Freiburg, Germany).

**Figure 5:**
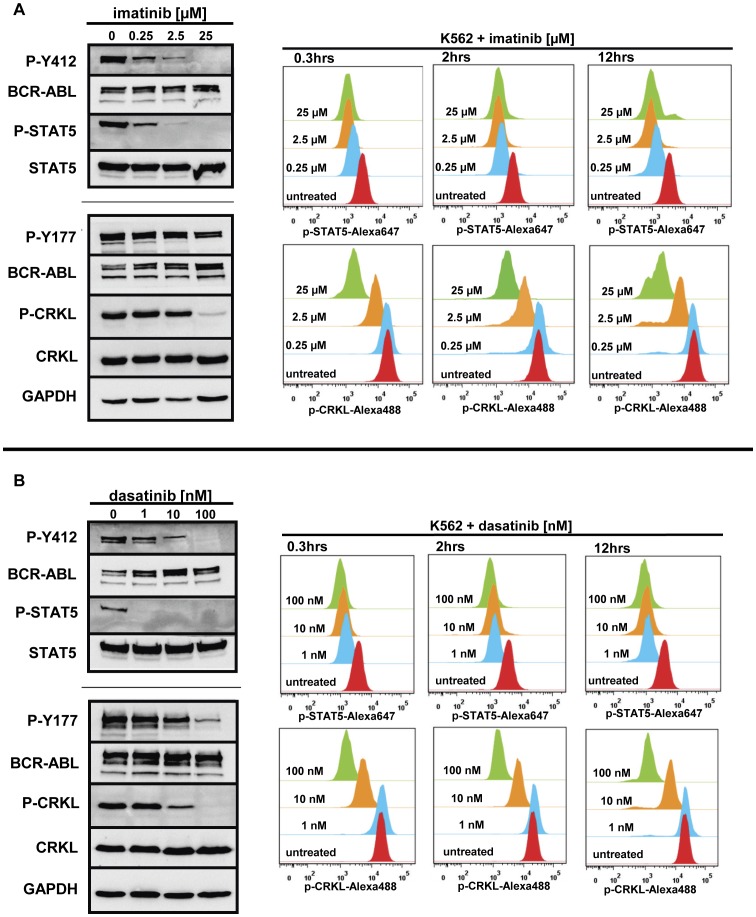
Kinetics and dynamics of BCR-ABL signaling upon tyrosine-kinase inhibition. K562 cells were treated with increasing concentrations of tyrosine kinase inhibitors. Untreated cells served as a control. For analysis of signaling pathway inhibition cells were either lysed for Western Blot analysis or fixed for FACS. (**A**): *Left panel*: Western Blot analysis of signaling pathway inhibition after exposure of Ba/F3-BCR-ABL cells to imatinib for 2 h. *Right panel*: FACS analysis of STAT5 and CRKL inhibition using phosphorylation-specific antibodies. To assess signaling dynamics, this analysis was performed at three different time-points (0.3, 2, and 12 h). (**B**) The same analysis was performed using the second-generation BCR-ABL tyrosine kinase inhibitor dasatinib. Experiments were performed at least in triplicate and one representative experiment is shown.

### Flow Cytometry

DNA content analysis was performed as described previously. [18] Briefly, cells were harvested, resuspended in 300 µl HFS-buffer (0,05 mg/ml propidium iodide, 1 mg/ml sodium-citrate, 1 µl/ml Triton X-100), and then subjected to FACS analysis. Annexin V staining was done using the PE Annexin V Apoptosis Detection Kit I (Becton Dickinson, Heidelberg, Germany) following the manufacturer’s protocol.

For intracellular staining of phospho-proteins and total-proteins 1×10^6^ cells were harvested and processed as described elsewhere. [19].

The following antibodies were used for staining: p-STAT5(Y694)-Alexa647, p-CRKL(Y207)-PE, ABCB1-FITC, ABCG2-PE (Becton Dickinson, Heidelberg, Germany), cleaved caspase3(asp715)-Alexa488 (Cell Signaling Technology, Danvers, MA, USA).

Flow cytometry analysis was done using FACSDiva-Software on a FACS Canto II cytometer (Becton Dickinson, Heidelberg, Germany). Final data analysis was performed using FlowJo Software (Tree Star, Ashland, Oregon, USA).

**Figure 6:**
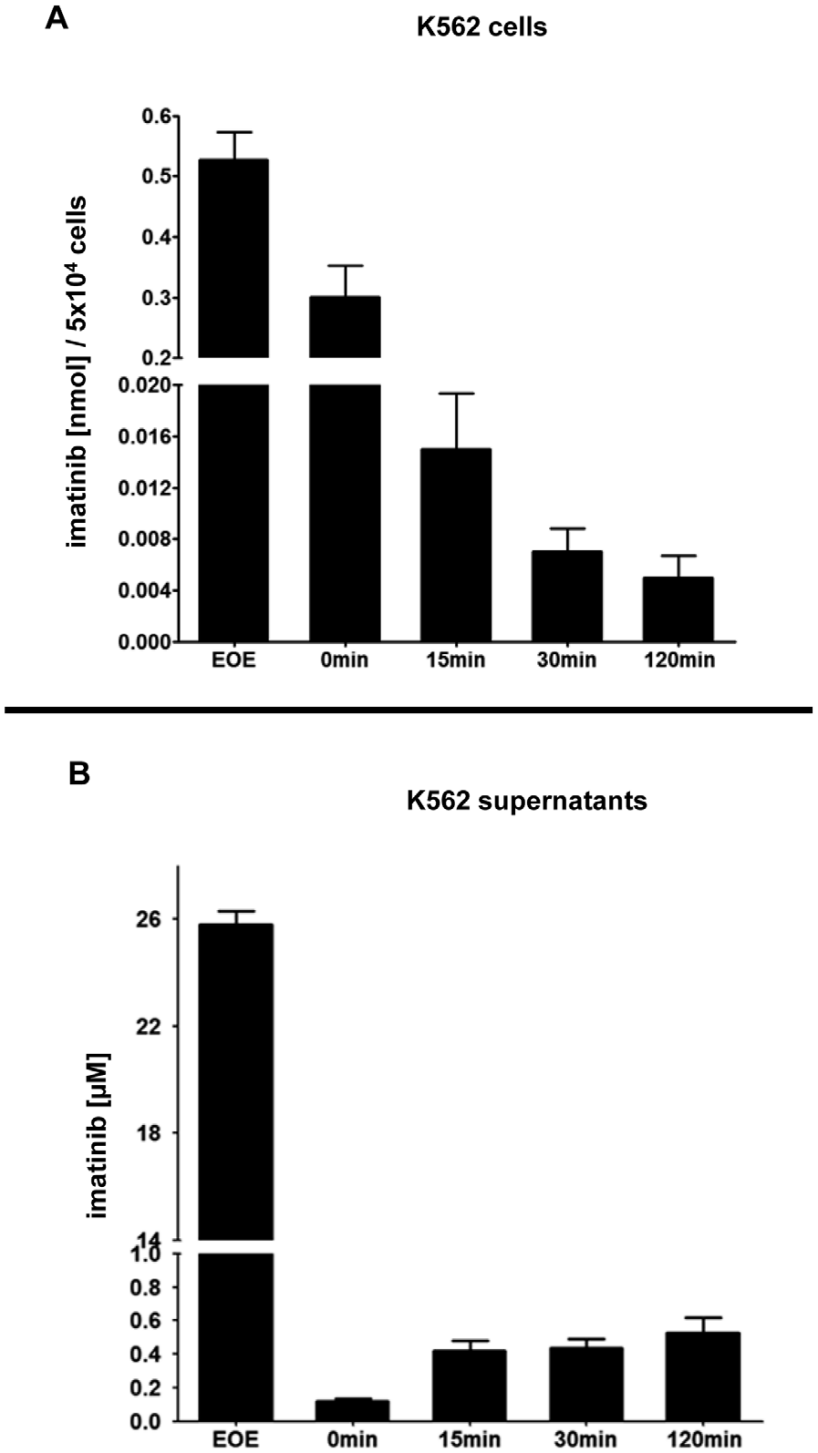
Measurement of cellular imatinib uptake and release upon HD-TKI pulse-exposure. 5×10^4^ K562 cells were treated for 2 h with 25 µM ^14^C-labeled imatinib in a total volume of 1 ml cell culture media followed by drug wash-out as described for **Figure 1B**. Specific activity of cell culture supernatants and cell pellets was measured using a scintillation counter. Imatinib concentrations were calculated by fitting the dpm (disintegrations per minute) values to a standard curve. All measurements were performed in triplicate. Depicted are mean values + SEM of 4 independent experiments. (**A**): Dynamics of cellular imatinib content was measured. Cells were pelleted and washed according to our drug wash-out protocol immediately at the end of the incubation period (end of exposure, “EOE”) and then subjected to scintillation counter assessment of cellular imatinib uptake. In parallel, for analysis of imatinib release, cells were replated in 1 ml fresh cell culture media and 0, 15, 30, and 120 min after replating cells were again pelleted, washed and then subjected to measurement in a scintillation counter. (**B**): Corresponding dynamics of imatinib concentration in cell culture supernatants upon HD-TKI pulse-exposure followed by drug wash-out. Measurement of cell culture supernatants immediately after 2 h incubation with 25 µM ^14^C-labeled imatinib represents the amount of imatinib present in the incubation medium (“EOE”). Intervals 0, 15, 30, and 120 min represent the time elapsed after initial drug wash-out and replating in fresh cell culture media.

### Radioactive Imatinib Uptake and Release Assay

For uptake of ^14^C-labeled imatinib (Novartis, Basel, Switzerland) 5×10^4^ cells/ml were incubated in the presence of 25 µM ^14^C-labeled imatinib for 2 h at 37°C. Cellular and aqueous phases were subsequently separated by centrifugation. The supernatant was prepared for analysis. The cell pellet was washed twice with 1 ml PBS/5×10^4^ cells as described above and samples were either subjected to replating in TKI-free cell culture media or prepared for analysis. Prior to measurement of intracellular imatinib concentration 5×10^4^ cells were washed twice with 1 ml PBS and subsequently resuspended into 1 ml of media and then mixed with 3 ml scintillation liquid (luma safe plus, Perkin Elmer, Rodgau, Germany). Specific activity of ^14^C-labeled imatinib was measured using a Packard TriCarb 1600TR liquid scintillation analyzer (Perkin Elmer, Rodgau, Germany). Concentrations of imatinib in cells and supernatants were calculated using a standard curve.

**Figure 7:**
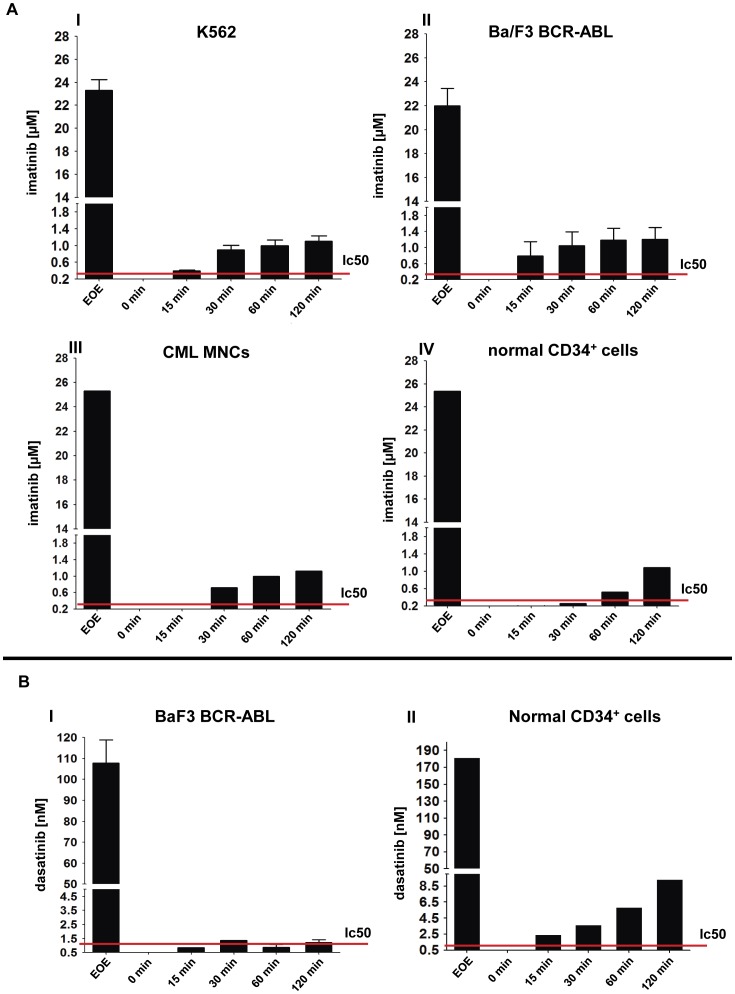
Measurement of TKI concentrations in cell culture supernatants using chromatographic methods. Imatinib and dasatinib concentrations were measured in cell culture supernatants using HPLC and LC/MS/MS, respectively. The sampling procedure and time-scale were identical to that described in **Figure 5**. (**A**) Ba/F3-BCR-ABL cells *(I)*, K562 cells *(II)*, primary human CML patient MNCs (*III*) and normal CD34^+^ enriched cells (*IV*) were pulse exposed to 25 µM imatinib followed by drug wash-out as described in **Figure 1B**. (**B**) Ba/F3-BCR-ABL cells (*I*) and normal enriched CD34^+^ cells (*II*) were pulse exposed to 100 nM dasatinib followed by drug wash-out as described in **Figure 1B**. The red lines represent the *in vitro* IC50 for the respective TKI used. Supernatant 1 (“S1”) was measured and served as a positive control. Measurement of cell culture media taken immediately upon replating served as a control for efficient washing (“0 min”). Depicted are mean values + SEM; at least 3 independent experiments were performed. For primary material one representative experiment is shown.

### HPLC Measurements of TKIs

An Extrelut ® NT 3 tube (Merck KGaA, Darmstadt, Germany) was used for the imatinib and midostaurin extraction from medium. 3 ml of a sample were added to the extraction tube and after 10 minutes the extraction tube was washed with 15 ml of diethyl-ether/ethyl-acetate (50∶50, v/v) and then with 15 ml of chloroform/isopropanol/ammonia (84∶15∶1, v/v/v). The solvents were collected in a glass reaction tube and evaporated until dry. The residue was redissolved in 200 µl of the mobile phase. For the dasatinib extraction from medium, the same method was used with the exception that a mixture of 2990 µl of a sample and 10 µl of 1 mg/l D-5 Fentanyl (internal standard) was added to the extraction tube.

Imatinib quantification in cell culture supernatants was performed using an Agilent HPLC 1200 Series system with a diode array detector operated at 265 nm.

For midostaurin the DAD was operated at 286 nm and the additional detection with a FLD was accomplished with excitation/emission wavelengths set at 286/386 nm. Data acquisition and integration was performed by ChemStation for LC 3D Systems. A Lichrospher 100-5 RP8, 250×4 mm column (Macherey-Nagel GmbH & Co. KG, Düren, Germany) maintained at 30±1°C for imatinib and at 40±1°C for midostaurin was used for the separation. 50 µl of a prepared sample were injected into the HPLC and eluted in a mobile phase of 0,05 M H3PO4/KH2PO4 - CH3CN (7∶3, v/v) for imatinib or of CH3OH-H2O-NH3 (89,7∶10∶0,3, v/v/v) for midostaurin at a flow rate of 1 ml/min.

**Figure 8:**
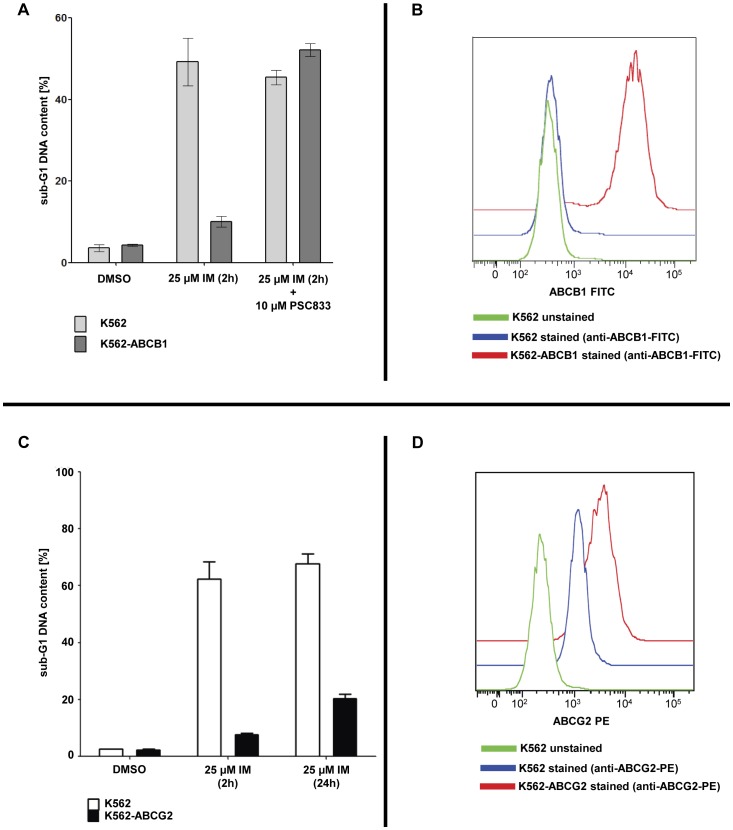
Overexpression of ABC transporters desensitizes K562 cells to high-dose imatinib pulse-exposure. (**A**) K562 and K562-ABCB1 cells were treated for 2 h with 25 µM imatinib either in the presence or absence of the ABCB1-inhibitor PSC833 Cells treated with the solvent DMSO served as negative controls. Sub-G1 DNA content analysis was performed 24 h after beginning of TKI exposure. The mean percentage of cells in sub-G1 phase ± SEM is depicted. Experiments were performed in triplicate. (**B**) K562 and K562-ABCB1 cells were surface stained with a FITC-labeled anti-ABCB1 antibody. Cells were analyzed by flow cytometry using a BD FACS Canto II cytometer. Overlay of measurements was performed using FlowJo Software (Tree Star, Ashland, Oregon, USA). (**C**) K562 and K562-ABCG2 cells were treated with 25 µM imatinib for 2 h, followed by drug wash-out. Cells were replated in fresh cell culture medium and samples were analyzed after 24 h by flow cytometry after propidium iodide staining. Untreated cells (“DMSO”) and continuously treated cells (“25 µM IM (24 h)”) served as controls. Three independent experiments were performed and the mean percentage of cells in subG1 phase + SEM is shown. (**D**) K562 and K562-ABCG2 cells were surface stained with a PE-labeled anti-ABCG2 antibody. Cells were analyzed by flow cytometry using a BD FACS Canto II cytometer. Overlay of measurements was performed using FlowJo Software (Tree Star, Ashland, Oregon, USA).

For dasatinib quantification an Agilent HPLC 1100 Series with a PE Sciex API 2000 LC/MS/MS System was used. The detector was operated in the MRM scan type with the 488.1/401.0 precursor/product ion for dasatinib and 342.4/188.2 for D-5 Fentanyl. Data acquisition and integration was performed by the Analyst 1.5 software. A Zorbax SB-C18, 4.6×50 mm, 5 micron column (Agilent Technologies Deutschland GmbH, Böblingen, Germany) maintained at 45±1°C was used for the separation. In total, 20 µl of a prepared sample were injected into the HPLC and eluted in a mobile phase consisting of A: CH_3_OH and B: 50 ml 0,1 M CH_3_COONH_4_+50 ml CH_3_CN +950 ml H_2_O +1 ml CH_3_COOH with a A:B ratio of 7∶3, v/v and a flow rate of 0,3 ml/min.

### Determination of Cell Size

Ba/F3-BCR-ABL cells were identified and photographed under a bright field fluorescence microscope (Axio Observer A1, Zeiss, Jena, Germany) and cell diameter was measured using MetaMorph® image analysis software. The mean diameter of 100 cells was used to calculate cellular volume, assuming that Ba/F3-BCR-ABL cells are spheric.

Intracellular imatinib concentration was calculated using the following formula:
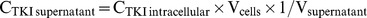



Intracellular TKI-concentration of a single cells can be calculated as follows:
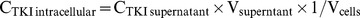



### Statistical Analyses

Data are presented as means + SEM if not otherwise stated.

## Results

### HD-TKI Pulse-exposure does not Alter Kinetics of Apoptosis

In previous studies, the underlying molecular mechanism for cell death upon high-dose TKI (HD-TKI) pulse-exposure remained elusive.[12]–[14] We speculated whether HD-TKI pulse-exposure may alter kinetics of cell death which would result in earlier onset of irreversible commitment to apoptosis. Thus, we treated BCR-ABL (p 210) expressing Ba/F3 cells (Ba/F3 BCR-ABL) with high-dose and conventional dose of either imatinib or dasatinib. Protein lysates were obtained at 0 h, 2 h, 4 h, 6, 8 h, 10 h, 12 h, and 24 h of treatment and monitored for caspase3 cleavage by immunoblotting. A rather uniform onset of caspase3 cleavage was observed after 6 to 8 h of TKI treatment, independent of TKI concentration or inhibitor used (see **Figure S1**). Thus, apparently, onset and kinetics of cell death program upon HD-TKI pulse-exposure was not altered. Therefore, it appeared to be unlikely that an alternative apoptotic pathway was activated by HD-TKI pulse-exposure.

### Repetitive Drug Wash-out Prevents Apoptotic Cell Death Upon High-dose TKI Pulse-exposure

To clarify, whether the wash-out regimen used affected drug efficacy, we set up experiments with serial drug wash-out procedures to observe the effects on cell death: Ba/F3 BCR-ABL cells and K562 cells (a CML blast crisis cell-line) were either treated with 25 µM imatinib or 100 nM dasatinib for 2 h followed by three consecutive rounds of thorough medium exchange (each consisted of 2 washing steps) at 2 h intervals. FACS analysis for apoptotic cell death was performed at 24 h (for details see **Figure 1**). In line with previously published data, apoptosis measured as subG1-DNA content (**Figure 2**), AnnexinV-positivity and caspase3 cleavage (**Figure 3**), respectively was efficiently induced by HD-TKI pulse-exposure in both cell-lines (**Figure 2** **and** **Figure 3** **,** upper panel: “1x”) while lower TKI concentrations did not result in apoptotic cell death upon pulse-exposure (see **Figure S2A**). As a control, off-target effects were ruled out by HD-TKI pulse-exposure of parental Ba/F3 cells with either imatinib or dasatinib (see **Figure S2B**). Induction of apoptosis after HD-TKI pulse-exposure could also be observed in the context of other oncogenes: Ba/F3 FLT3-ITD cells and MV 4–11 cells treated with midostaurin and Ba/F3 JAK2V617F cells treated with JAK-Inhibitor I showed similar results (data not shown). This further confirmed HD-TKI pulse-exposure induced apoptosis as being target specific and independent of cellular background.

Pulse-exposure using HD-TKI was previously reported to irreversibly induce cell death. However, unexpectedly, when cells were extensively washed a second (2 h post the initial media exchange) and a third time (4 h post initial media exchange) almost all cells survived HD-TKI pulse treatment and were rescued from apoptotic cell death (**Figure 2** **and** **Figure 3**, upper panel: “2x” & “3x”; **Figure S3A+B**). Moreover, incubation of previously untreated cells with cell culture supernatants derived from the second (S2) media exchange resulted in substantial increase in the fraction of apoptotic cells (**Figure 2** **and** **Figure 3**, lower panel: “S2”; **Figure S3A+B**). Similar results were obtained using FLT3-ITD or JAK2V617F expressing cell-lines (Ba/F3 FLT3-ITD, MV4-11 and Ba/F3 JAK2V617F) which were treated with midostaurin or JAK-inhibitor I, respectively (data not shown).

Since we employed a drug wash-out protocol that was slightly different from previously published experimental procedures, as a control, we performed a set of experiments following the drug wash-out procedure published by Shah and colleagues. [12] Employing this wash-out protocol repetitively (three times every 2 hours), we were able to confirm our findings as described above (**Figure S3A+B**). These results were surprising as the first extensive drug wash-out should have removed all biologically active TKI. This raised the possibility that intracellular TKI retention, leakage into the medium over time and thus prolonged presence of biologically active TKI concentrations might be responsible for induction of cell death upon HD-TKI pulse-exposure.

### HD-TKI Pulse-exposure Results in Prolonged Inhibition of Intracellular Signaling Nodes

To test for residual TKI activity, we then investigated the kinetics of inhibition of key signaling nodes (BCR-ABL, STAT5 and CRKL) in response to HD-TKI pulse-exposure. HD-TKI exposure results in early dephosphorylation of BCR-ABL (Y412), STAT5 (Y694), and CRKL (Y207), while BCR-ABL (Y177) did not show significant dephosphorylation after 2 h TKI exposure (**Figure 4A**, lane 8, “2 h”). After the first round of drug wash-out (lane 5, “1x”), STAT5 signaling was still suppressed, while CRKL appeared to be largely rephosphorylated again. These effects were seen with both imatinib and dasatinib. Using imatinib, analysis of BCR-ABL phosphorylation (Y412) showed strong inhibition of autophosphorylation and BCR-ABL (Y412) remained dephosphorylated after the first round of drug wash-out, while this was less evident using dasatinib (**Figure 4A**, “1x”). To achieve better quantitation of signaling events upon drug wash-out, we performed multiparameter intracellular flow cytometry using phospho-specific antibodies against CRKL and STAT5. These experiments confirmed our western blot data, demonstrating strong dephosphorylation of STAT5 and only slight dephosphorylation of CRKL after the first drug wash-out upon HD-TKI pulse-exposure (**Figure 4B** **,** “1x”).

Recovery of STAT5-phosphorylation levels correlated well with rescue from apoptotic cell death: We observed gradual reappearance of STAT5-phosphorylation signals after the second round of drug wash-out (“2x”) reaching complete recovery of phosphorylation signals in all cell-lines tested upon the third round (“3x”) of media exchange (**Figure 4B**). At the same time, the proportion of apoptotic cells decreased accordingly (**Figure 2** **and** **Figure 3**).

As an internal control, incubation of previously untreated cells with supernatants derived from the corresponding washing steps 1 (S1), 2 (S2) and 3 (S3) perfectly mirrored the results observed in washed cells: phosphorylation signals in S1 resemble those after 2 h TKI treatment, phosphorylation signals in S2 resemble those in “1x”, and phosphorylation signals in S3 resemble those in “2x” (**Figure 4A**). These data could also be confirmed independently using K562 cells (**Figure S4A**). Again, observation of biological activity in supernatants 2 (S2) and 3 (S3) was surprising as the first extensive washing procedure should have removed all remaining biologically active TKI. This indicates an abundance of active TKIs in supernatants obtained after drug wash-out. Thus, in line with data presented above, these results suggest intracellular retention of TKIs which is released into the re-plating media over time.

### BCR-ABL Signaling Nodes Exhibit Different Kinetics upon TKI Exposure

Next, we investigated biochemical dose response of phospho-tyrosine residues of BCR-ABL (Y412 and Y177), CRKL and STAT5 in Ba/F3-BCR-ABL and K562 cells upon TKI exposure (**Figure 5** and **Figure S4B**). Using Western blotting and intracellular phospho-flow analysis, we observed a rapid downregulation of STAT5-phosphorylation after 20 min exposure to low TKI concentrations (0.25 µM imatinib or 1 nM dasatinib) with complete inhibition of STAT5-phosphorylation occurring at higher concentrations (**Figure 5**). At the same time, CRKL-phosphorylation remained almost unchanged following exposure to low imatinib (0.25 µM) or dasatinib (1 nM) concentrations (**Figure 5**). A slight decrease in CRKL-phosphorylation was observed upon exposure to 2.5 µM imatinib or 10 nM dasatinib. Complete dephosphorylation of CRKL was achieved upon exposure to very high concentrations of imatinib (25 µM) or dasatinib (100 nM) only (**Figure 5**). When investigating later time-points (2 h and 12 h), we did not observe obvious changes in CRKL-phosphorylation levels as compared to the 0.3 h time-point (**Figure 5**). Looking at BCR-ABL-phosphorylation itself, we confirmed our previous observation that BCR-ABL (Y412) behaves similarly to STAT5, while BCR-ABL (Y177) exhibits only minor phosphorylation changes at least at the 2 h time-point examined by Western blot in this study (**Figure 5**). In summary, our data indicate that suppression of BCR-ABL kinase activity, as measured by dephosphorylation of BCR-ABL (Y412) and STAT5 (Y694), does not necessarily result in immediate suppression of either CRKL (Y207)- or BCR-ABL (Y177)-phosphorylation. Thus, in our hands, the phosphorylation status of CRKL and BCR-ABL (Y177) did not correlate well with the presence or absence of biologically active TKI concentrations. Taken together, these results suggest that dephosphorylation of CRKL and BCR-ABL (Y177) are inadequate surrogates for inhibition of BCR-ABL kinase activity in the setting of experimental drug wash-out.

### TKIs Show Intracellular Accumulation and are Released in a Time-dependent Fashion upon HD-TKI Pulse-exposure

The findings described above suggest that the effects observed upon HD-TKI pulse-exposure are attributable to intracellular TKI retention resulting in prolonged inhibition of intracellular signaling pathways. To confirm whether redistribution of TKIs from pulse-treated cells into the re-plating medium occurs, TKI concentrations (imatinib and dasatinib) were analyzed intracellularly and in cellular supernatants.

As an initial approach, we treated K562 cells with 25 µM ^14^C-labeled imatinib. Measurement of the cellular phase immediately after our standard drug wash-out protocol (**Figure 6A**, “EOE”) revealed a dramatic cellular drug uptake: given a mean cellular volume of 2.487×10^−12^ L/cell (see **Table S1B**), the intracellular imatinib concentration (based on “EOE”, **Figure 6A**) was calculated to be about 4.2 mM. We then re-plated the cells in cell culture medium and subsequently measured intracellular and extracellular imatinib concentrations simultaneously at predefined time-points (0, 15, 30, and 120 min). This resulted in almost complete removal of imatinib in the extracellular compartment (time-point 0 min, **Figure 6B**), while the intracellular drug concentration remained relatively high (time-point 0 min, **Figure 6A**). Importantly, we then observed a time-dependent increase of extracellular imatinib concentration reaching a mean final concentration of 0.53±0.18 µM at 120 min after drug wash-out (**Figure 6B**). This was paralleled by a time-dependent decrease in cellular imatinib concentration (**Figure 6A**). Thus, these findings indeed demonstrated significant TKI release from cells into the medium. To control for technical quality of drug wash-out, we performed up to 4 serial washing steps and measured imatinib concentration in cell culture supernatants immediately after resuspension of cells: We found that increasing washing steps beyond 2 did not result in further relevant decrease of imatinib concentration (see **Figure S5**). Imatinib remained detectable even after as much as 4 washing steps suggesting continuous drug release from intracellular reservoirs.

Next, we set up an HPLC-based method for measurement of extracellular and intracellular TKIs to be able to measure different TKIs without being dependent on the availability of isotope labeled TKIs. First, we measured imatinib in cell culture supernatants and cellular lysates to test whether it is technically feasible to confirm the results obtained with radioactively labeled imatinib. We found close correlation between HPLC-data and those obtained using ^14^C-labeled imatinib (**Figure 7A** **, panel I** and **Table S1A**). To test whether this mechanism is also operative in an independent BCR-ABL driven cell model and in primary human cells, we then investigated imatinib release upon HD-TKI pulse-exposure using different cell populations: Ba/F3-BCR-ABL cells as well as mononuclear cells from untreated CML patients and normal CD34^+^ hematopoietic progenitor cells from healthy donors exhibited the same kinetics as previously demonstrated for K562 cells (**Figure 7A** **, panels II–IV**). Next, we evaluated a method for extracellular and intracellular detection of dasatinib. Similarly to imatinib, dasatinib exhibited massive intracellular accumulation as well as slow time-dependent drug release into cell culture media following drug wash-out. In Ba/F3-BCR-ABL cells, dasatinib reached a final mean concentration of 1.22 nM in the extracellular media 2 h after drug wash-out (**Figure 7B** **, panel I**). This is well above the reported *in vitro* IC_50_ level of 0.5–1 nM. [20], [21] A similar result with even higher dasatinib levels was obtained in normal CD34^+^ cells from healthy donors (**Figure 7B** **, panel II**).

Together, these findings provide experimental evidence that biologically active TKI is retained intracellularly upon HD-TKI pulse-exposure in BCR-ABL positive cell-lines and in primary human cells.

### Overexpression of ABCB1 Prevents Apoptosis upon Imatinib Pulse-exposure

ABC-family transporters have been reported to be involved in active TKI efflux and TKI resistance.[22]–[24] For imatinib, it has been shown that ABCB1 and ABCG2 play a major role in drug efflux-mediated drug resistance.[24]–[28] If intracellular TKI accumulation and retention indeed mediates apoptosis upon HD-TKI pulse-exposure, we hypothesized that ABC-transporters may prevent cells from apoptosis upon HD-TKI pulse-exposure. Indeed, overexpression of either ABCB1 or ABCG2 in K562 cells resulted in prevention of apoptotic cell death upon HD-imatinib pulse-exposure (**Figure 8**). Addition of the ABCB1-specific inhibitor PSC833 restored susceptibility to HD-imatinib pulse-exposure (**Figure 8** **A**), while treatment of K562-ABCB1 cells with PSC833 alone did not result in any significant induction of apoptosis (see **Figure S6)**.

## Discussion

Induction of apoptosis upon HD-TKI pulse-exposure has been demonstrated by several groups.[12]–[14] Based upon these findings, a concept of irreversible commitment to apoptosis upon HD-TKI pulse-exposure was proposed. However, the mechanism of induction of apoptosis upon HD-TKI pulse-exposure remained elusive at the molecular level. [12] This prompted us to investigate the molecular mechanisms of cell death induced by HD-TKI pulse-exposure in more detail. It appeared unlikely that short-term potent kinase inhibition could initiate an irreversible cell death program without altering onset and kinetics of apoptosis. Indeed, the data presented here provide evidence that HD-TKI pulse-exposure does not irreversibly initiate apoptosis, since cells can be completely rescued by drug wash-out. Western blot experiments confirmed persistent target inhibition after HD-TKI pulse-exposure, with no evidence of BCR-ABL (Y412) or STAT5 re-phosphorylation after the first round of media exchange. This indicates substantial residual TKI activity when employing a single drug wash-out procedure.

In line with previously published data on HD-TKI pulse-exposure, we observed re-phosphorylation of CRKL in BCR-ABL cells after the first drug wash-out step, while discordantly BCR-ABL (Y412) and STAT5 were still dephosphorylated.[12]–[14] Interestingly, BCR-ABL (Y177)-phosphorylation remained almost unaffected upon TKI exposure. This suggested differential kinetics and/or dynamics of BCR-ABL (Y412)- and STAT5-phosphorylation as compared to CRKL- and BCR-ABL (Y177)-phosphorylation. Employing titration experiments using increasing concentrations of either imatinib or dasatinib, we measured STAT5- and CRKL-phosphorylation after different incubation times. This confirmed different kinetics as well as dynamics of STAT5- versus CRKL-phosphorylation. This difference might translate into a low diagnostic sensitivity for residual TKI activity *in vitro*, if CRKL-phosphorylation is used as a sole test for BCR-ABL tyrosine kinase activity. [12]–[14].

The apparent contradiction in our finding, that BCR-ABL (Y177)-phosphorylation does not correlate with BCR-ABL substrate phosphorylation is supported by recent publications. [29] While BCR-ABL (Y177) has been shown to play a crucial role for leukemic transformation capacity of BCR-ABL[30]–[33], kinase activity of BCR-ABL and downstream signaling is mainly regulated by BCR-ABL (Y412)-phosphorylation.[34]–[36] Along this line, a recent paper demonstrated that BCR-ABL (Y177) is phosphorylated by JAK2, and not by ABL. [29] It has been proposed that BCR-ABL (Y177)-phosphorylation provides fine-tuning of BCR-ABL downstream signaling rather than switching BCR-ABL signaling on and off. [29] In our hands, STAT5 is a useful surrogate parameter to monitor immediate effects of BCR-ABL kinase activity as its phosphorylation positively correlates with cell survival. Moreover, it has been demonstrated that STAT5 signaling is indispensable for initiation and maintenance of BCR-ABL mediated leukemic transformation.[37]–[40].

Results obtained by employing successive rounds of drug wash-out suggested prolonged intracellular TKI exposure to be the critical mechanism involved in induction of apoptosis upon HD-TKI pulse-treatment. Dose-dependent intracellular accumulation of TKI upon imatinib exposure has already been reported previously. [41] Along this line, we hypothesized that pronounced intracellular TKI-accumulation might be responsible for the observed results. Indeed, measurements of intracellular imatinib and dasatinib uncovered remarkable intracellular TKI accumulation upon HD-TKI pulse-exposure. Moreover, intracellular TKI accumulation is characterized by a slow time-dependent decrease in intracellular TKI levels upon drug wash-out. This was paralleled by a time-dependent increase of TKI concentrations in extracellular media upon washing and re-plating of cells, indicating release of TKI from an intracellular compartment into the cell culture media. Consistent with this, we demonstrated that HD-TKI pulse-exposure with imatinib was ineffective at inducing apoptosis in cells expressing the ABC-family transporters ABCB1 or ABCG2. Of note, sensitivity to HD-TKI pulse-exposure was restored by pharmacological inhibition of ABC-transporters.

From a clinical perspective, our findings may prove useful for refinement of effective TKI dosing schedules, especially when applying TKIs with short plasma half-life. Along this line, recently, it was demonstrated that a short plasma half-life of a given compound may not necessarily predict a deficit in terms of clinical efficacy. [10] Our *in vitro* model of HD-TKI pulse-exposure revealed a previously unrecognized pharmacokinetic interplay between TKI concentrations in the extracellular media and intracellular TKI concentrations when a high-dose TKI pulse is applied. Both, dramatic intracellular TKI accumulation and delayed TKI release strongly argue in favor of an active cellular maintenance and/or uptake mechanism that can prevent a sudden decrease in intracellular TKI concentration. Indeed, recently it has been demonstrated that OCT-1 mediates cellular influx of imatinib, and that transporter activity correlates with efficacy.[42]–[44] On the other hand, it has been shown that OCT-1 has less impact on cellular influx of dasatinib and nilotinib. [43], [45], [46] Therefore, we believe that additional drug-transporter proteins contribute to intracellular accumulation of TKIs. However, the data presented here is consistent with a model where intracellular accumulation and retention of TKIs *in vivo* also translates into significantly higher intracellular TKI concentrations as compared to the extracellular medium. It is conceivable that in the setting of high-dose pulse therapy this may then result in prolonged intracellular TKI exposure significantly exceeding plasma half-life of a given TKI.

In conclusion, we show that dramatic intracellular TKI accumulation and retention result in prolonged target inhibition which appears to be the sole underlying molecular mechanism in HD-TKI pulse-exposure (imatinib, dasatinib) mediated induction of apoptosis *in vitro*. Moreover, the data illustrate that potent but transient kinase inhibition per se is not sufficient to irreversibly commit oncogene transformed cells to apoptosis. As we have observed intracellular TKI accumulation and retention in other oncogenic kinase models such as FLT3-ITD [47] and JAK2-V617F (data not shown), the mechanism described here may indicate a general pharmacokinetic feature of TKIs. However, this point clearly requires further investigation.

Based on our data presented here, monitoring both, plasma and intracellular drug levels of imatinib and dasatinib *in vivo* will provide pharmacokinetic data which may prove useful to optimize dosing schedules in upcoming clinical trials. We speculate that either the design of inhibitors that accumulate and are retained in target cells or, alternatively, co-administration of drugs which result in intracellular enrichment of specific TKIs may improve TKI therapy in the future.

## Supporting Information

Figure S1 **Onset of apoptosis is unchanged upon exposure to high TKI concentrations.** Ba/F3-BCR-ABL cells were treated using different TKI concentrations. Cells were lysed at different time-points using lysis buffer and prepared for Western blot analysis. Immunoblotting was performed using monoclonal antibodies specific for total caspase3 and cleaved caspase3. To control for equal loading, blots were stripped and re-probed with anti-actin antibody. (**A**) depicts results obtained using imatinib (**B**) depicts results obtained using dasatinib. At least two independent experiments were performed and one representative experiment is shown.
(PDF)

Figure S2 **Control cells do not reveal significant cytotoxic effects upon HD-TKI pulse exposure. (A)** Ba/F3-BCR-ABL cells (5×10^4^ cells/ml, total volume 2 ml) were treated with TKI as indicated for 2 h followed by extensive drug wash-out using 2×2 ml PBS. Cells were then re-seeded in 2 ml cell culture medium without TKI. Cells exposed to 0.35% DMSO served as controls (“0 h”). Cells continuously exposed to TKI served as positive controls (“24 h”). Twenty-four hours after start of TKI exposure the percentage of cells in subG1 phase was measured by flow cytometry after propidium iodide staining. Three independent experiments were performed. Data are presented as mean percentage of cells in subG1 phase + SEM. (**B**) Ba/F3 parental cells (5×10^4^ cells/ml, total volume 2 ml) were treated for 2 h with TKI as indicated followed by thorough drug wash-out using 2×2 ml PBS. Cells were then reseeded in 2 ml cell culture medium without TKI. Twenty-four hours after start of TKI exposure the percentage of cells in subG1 phase was measured by flow cytometry after propidium iodide staining. At least three independent experiments were performed and data are presented as mean percentage of cells in subG1 phase + SEM.
(PDF)

Figure S3 **Repetitive washing prevents apoptosis in K562 cells –effect of a different wash-out protocol.** K562 cells were treated either with imatinib or dasatinib as indicated. To control for the effects of different washing protocols, in this case the wash-out procedure was performed as previously described by Shah et al. 2008. In brief, cells (5×10^4^ cells/ml, total volume 2 ml for PI staining and 20 ml for AnnexinV and cleaved caspase3 staining) were washed three times with a volume of medium (containing 10% FCS) that consisted of 50% of the volume of the drug exposure. Cells were afterwards replated in fresh medium (+10% FCS) without inhibitor. For repetitive washing procedures under the same conditions, we generally followed the scheme as is depicted in **Figure 1B**. (**A**) Results of PI measurement of cells at 48 hours. Three independent experiments were performed and data are presented as mean percentage of cells in subG1 phase + SEM. (**B**) FACS measurement of AnnexinV and cleaved caspase3 at 48 hours. The Y-axis represents forward scatter (linear scale) and the X-axis depicts the signal intensity of AnnexinV (left) and cleaved caspase3 (right) on a log-scale. Three independent experiments were performed. One representative experiment is shown.
(PDF)

Figure S4 **Intracellular signaling in K562 cells upon HD-TKI exposure –effect of a different wash-out protocol.** K562 cells (5×10^4^ cells/ml, total volume 20 ml) were treated with indicated TKI concentrations. Wash-out was performed as previously described by Shah et al. 2008. In brief, cells were washed three times with medium containing 10% FCS with a volume of medium that consisted of 50% of the volume of the drug exposure. Cells were afterwards replated in fresh medium (+10% FCS) without inhibitor. For repetitive washing procedures under the same conditions we generally followed the scheme as is depicted in **Figure 1B**. (**A**) Western Blot analysis of important signaling downstream nodes. Samples were lysed 2 h after each washing step. Untreated cells served as positive controls for phosphorylation signals. Cells treated continuously with TKI for 2 hours or 10 hours (“2 h” and “10 h”) served as positive controls for TKI activity. (**B**) Cells were treated for 2 h with 100 nM dasatinib, followed by serum wash-out. At various time points after wash-out cells were lysed and prepared for western blot analysis. Phosphotyrosine content was determined using the phosphotyrosine antibodies Y100 and 4G10 as well as P-BCR-ABL (Y177) and (Y412). Antibodies against ABL and GAPDH served as loading control.
(PDF)

Figure S5 **Determination of washing efficiency.** K562 cells (5×10^4^/ ml) were pulse exposed for 2 h with 25 µM ^14^C-labeled imatinib followed by wash-out with PBS (1 ml per 5×10^4^ cells per washing step). Immediately after each washing step the PBS supernatant was subjected to beta-counter analysis to measure the concentration of remaining imatinib. After 4 washing steps, cells were replated into TKI free media. Imatinib concentration was then measured 2 h after the last washing step (“+120”). Supernatant analyzed at the end of the TKI exposure (“EOE”) represented a positive control for applied TKI. All measurements were performed in triplicate. Depicted are mean values + SEM of 3 independent experiments. Imatinib concentrations were calculated by fitting the dpm values to a standard curve.
(PDF)

Figure S6 **ABCB1 expression confers imatinib resistance in K562 cells.** K562 and K562-ABCB1 cells were treated continuously either with 0.5 µM or 25 µM imatinib in the presence or absence of 10 µM PSC833 as indicated. Cells exposed to 0.35% DMSO or 10 µM PSC833 alone served as a control. Samples were analyzed after 24 h by flow cytometry after propidium iodide staining. Three independent experiments were performed and results are shown as means ± SEM.
(PDF)

Table S1 **TKI concentrations measured by HPLC/LC-MS/MS in cellular lysates.** (**A**) TKI concentrations ± SEM measured by HPLC/LC-MS/MS in cellular lysates treated with 25 µM imatinib or 100 nM dasatinib for 2 h as indicated followed by drug wash-out. Intracellular TKI concentrations were calculated based on the mean cellular volume. (**B**) Mean size in µm ± SD of Ba/F3-BCR-ABL cells and K562 cells. In total, 100 cells of each cell line were measured. Intracellular imatinib concentration was calculated based on the assumption that cells are spherical (V = 4/3×π×r^3^). Thus, the total cellular volume for 0.2×10^6^ cells is 0.19 µl for Ba/F3-BCR-ABL cells and 0.5 µl for K562 cells.
(PDF)

## References

[1] Hochhaus A, O’Brien SG, Guilhot F, Druker BJ, Branford S (2009). Six-year follow-up of patients receiving imatinib for the first-line treatment of chronic myeloid leukemia.. Leukemia.

[2] Kindler T, Lipka DB, Fischer T (2010). FLT3 as a therapeutic target in AML: still challenging after all these years.. Blood.

[3] Verstovsek S, Kantarjian H, Mesa RA, Pardanani AD, Cortes-Franco J (2010). Safety and efficacy of INCB018424, a JAK1 and JAK2 inhibitor, in myelofibrosis.. N Engl J Med.

[4] Pardanani A, Gotlib JR, Jamieson C, Cortes JE, Talpaz M (2011). Safety and efficacy of TG101348, a selective JAK2 inhibitor, in myelofibrosis.. J Clin Oncol.

[5] le Coutre P, Mologni L, Cleris L, Marchesi E, Buchdunger E (1999). In vivo eradication of human BCR/ABL-positive leukemia cells with an ABL kinase inhibitor.. J Natl Cancer Inst.

[6] Larson RA, Druker BJ, Guilhot F, O’Brien SG, Riviere GJ (2008). Imatinib pharmacokinetics and its correlation with response and safety in chronic-phase chronic myeloid leukemia: a subanalysis of the IRIS study.. Blood.

[7] Picard S, Titier K, Etienne G, Teilhet E, Ducint D (2007). Trough imatinib plasma levels are associated with both cytogenetic and molecular responses to standard-dose imatinib in chronic myeloid leukemia.. Blood.

[8] Druker BJ, Talpaz M, Resta DJ, Peng B, Buchdunger E (2001). Efficacy and safety of a specific inhibitor of the BCR-ABL tyrosine kinase in chronic myeloid leukemia.. N Engl J Med.

[9] Talpaz M, Shah NP, Kantarjian H, Donato N, Nicoll J (2006). Dasatinib in imatinib-resistant Philadelphia chromosome-positive leukemias.. N Engl J Med.

[10] Shah NP, Kantarjian HM, Kim DW, Rea D, Dorlhiac-Llacer PE (2008). Intermittent target inhibition with dasatinib 100 mg once daily preserves efficacy and improves tolerability in imatinib-resistant and -intolerant chronic-phase chronic myeloid leukemia.. J Clin Oncol.

[11] Shah NP, Kim DW, Kantarjian H, Rousselot P, Llacer PE (2010). Potent, transient inhibition of BCR-ABL with dasatinib 100 mg daily achieves rapid and durable cytogenetic responses and high transformation-free survival rates in chronic phase chronic myeloid leukemia patients with resistance, suboptimal response or intolerance to imatinib.. Haematologica.

[12] Shah NP, Kasap C, Weier C, Balbas M, Nicoll JM (2008). Transient potent BCR-ABL inhibition is sufficient to commit chronic myeloid leukemia cells irreversibly to apoptosis.. Cancer Cell.

[13] Snead JL, O’Hare T, Adrian LT, Eide CA, Lange T (2009). Acute dasatinib exposure commits Bcr-Abl-dependent cells to apoptosis.. Blood.

[14] Hiwase DK, White DL, Saunders VA, Kumar S, Melo JV (2009). Short-term intense Bcr-Abl kinase inhibition with nilotinib is adequate to trigger cell death in BCR-ABL(+) cells.. Leukemia.

[15] Markova B, Albers C, Breitenbuecher F, Melo JV, Brummendorf TH (2010). Novel pathway in Bcr-Abl signal transduction involves Akt-independent, PLC-gamma1-driven activation of mTOR/p70S6-kinase pathway.. Oncogene.

[16] Marie JP, Faussat-Suberville AM, Zhou D, Zittoun R (1993). Daunorubicin uptake by leukemic cells: correlations with treatment outcome and mdr1 expression.. Leukemia.

[17] Zhou S, Zong Y, Ney PA, Nair G, Stewart CF (2005). Increased expression of the Abcg2 transporter during erythroid maturation plays a role in decreasing cellular protoporphyrin IX levels.. Blood.

[18] Kindler T, Breitenbuecher F, Kasper S, Stevens T, Carius B (2003). In BCR-ABL-positive cells, STAT-5 tyrosine-phosphorylation integrates signals induced by imatinib mesylate and Ara-C.. Leukemia.

[19] Irish JM, Hovland R, Krutzik PO, Perez OD, Bruserud Ø (2004). Single Cell Profiling of Potentiated Phospho-Protein Networks in Cancer Cells.. Cell.

[20] Shah NP, Tran C, Lee FY, Chen P, Norris D (2004). Overriding imatinib resistance with a novel ABL kinase inhibitor.. Science.

[21] Lombardo LJ, Lee FY, Chen P, Norris D, Barrish JC (2004). Discovery of N-(2-chloro-6-methyl- phenyl)-2-(6-(4-(2-hydroxyethyl)- piperazin-1-yl)-2-methylpyrimidin-4- ylamino)thiazole-5-carboxamide (BMS-354825), a dual Src/Abl kinase inhibitor with potent antitumor activity in preclinical assays.. J Med Chem.

[22] Jordanides NE, Jorgensen HG, Holyoake TL, Mountford JC (2006). Functional ABCG2 is overexpressed on primary CML CD34+ cells and is inhibited by imatinib mesylate.. Blood.

[23] Zhou S, Schuetz JD, Bunting KD, Colapietro AM, Sampath J (2001). The ABC transporter Bcrp1/ABCG2 is expressed in a wide variety of stem cells and is a molecular determinant of the side-population phenotype.. Nat Med.

[24] Deenik W, van der Holt B, Janssen JJ, Chu IW, Valk PJ (2010). Polymorphisms in the multidrug resistance gene MDR1 (ABCB1) predict for molecular resistance in patients with newly diagnosed chronic myeloid leukemia receiving high-dose imatinib.. Blood 116: 6144–6145; author reply 6145–6146.

[25] Mahon FX, Belloc F, Lagarde V, Chollet C, Moreau-Gaudry F (2003). MDR1 gene overexpression confers resistance to imatinib mesylate in leukemia cell line models.. Blood.

[26] Brendel C, Scharenberg C, Dohse M, Robey RW, Bates SE (2007). Imatinib mesylate and nilotinib (AMN107) exhibit high-affinity interaction with ABCG2 on primitive hematopoietic stem cells.. Leukemia.

[27] Ni LN, Li JY, Miao KR, Qiao C, Zhang SJ (2011). Multidrug resistance gene (MDR1) polymorphisms correlate with imatinib response in chronic myeloid leukemia.. Med Oncol.

[28] Illmer T, Schaich M, Platzbecker U, Freiberg-Richter J, Oelschlagel U (2004). P-glycoprotein-mediated drug efflux is a resistance mechanism of chronic myelogenous leukemia cells to treatment with imatinib mesylate.. Leukemia.

[29] Samanta A, Perazzona B, Chakraborty S, Sun X, Modi H (2011). Janus kinase 2 regulates Bcr-Abl signaling in chronic myeloid leukemia.. Leukemia.

[30] Chu S, Li L, Singh H, Bhatia R (2007). BCR-tyrosine 177 plays an essential role in Ras and Akt activation and in human hematopoietic progenitor transformation in chronic myelogenous leukemia.. Cancer Res.

[31] Pendergast AM, Gishizky ML, Havlik MH, Witte ON (1993). SH1 domain autophosphorylation of P210 BCR/ABL is required for transformation but not growth factor independence.. Mol Cell Biol.

[32] Nieborowska-Skorska M, Wasik MA, Slupianek A, Salomoni P, Kitamura T (1999). Signal transducer and activator of transcription (STAT)5 activation by BCR/ABL is dependent on intact Src homology (SH)3 and SH2 domains of BCR/ABL and is required for leukemogenesis.. J Exp Med.

[33] Sattler M, Mohi MG, Pride YB, Quinnan LR, Malouf NA (2002). Critical role for Gab2 in transformation by BCR/ABL.. Cancer Cell.

[34] Pluk H, Dorey K, Superti-Furga G (2002). Autoinhibition of c-Abl.. Cell.

[35] Dorey K, Engen JR, Kretzschmar J, Wilm M, Neubauer G (2001). Phosphorylation and structure-based functional studies reveal a positive and a negative role for the activation loop of the c-Abl tyrosine kinase.. Oncogene.

[36] Brasher BB, Van Etten RA (2000). c-Abl has high intrinsic tyrosine kinase activity that is stimulated by mutation of the Src homology 3 domain and by autophosphorylation at two distinct regulatory tyrosines.. J Biol Chem.

[37] Scherr M, Chaturvedi A, Battmer K, Dallmann I, Schultheis B (2006). Enhanced sensitivity to inhibition of SHP2, STAT5, and Gab2 expression in chronic myeloid leukemia (CML).. Blood.

[38] Hoelbl A, Kovacic B, Kerenyi MA, Simma O, Warsch W (2006). Clarifying the role of Stat5 in lymphoid development and Abelson-induced transformation.. Blood.

[39] Hoelbl A, Schuster C, Kovacic B, Zhu B, Wickre M (2010). Stat5 is indispensable for the maintenance of bcr/abl-positive leukaemia.. EMBO Mol Med.

[40] Walz C, Ahmed W, Lazarides K, Betancur M, Patel N (2012). Essential role for Stat5a/b in myeloproliferative neoplasms induced by BCR-ABL1 and Jak2V617F in mice.. Blood.

[41] le Coutre P, Kreuzer KA, Pursche S, Bonin M, Leopold T (2004). Pharmacokinetics and cellular uptake of imatinib and its main metabolite CGP74588.. Cancer Chemother Pharmacol.

[42] Thomas J, Wang L, Clark RE, Pirmohamed M (2004). Active transport of imatinib into and out of cells: implications for drug resistance.. Blood.

[43] White DL, Saunders VA, Dang P, Engler J, Zannettino AC (2006). OCT-1-mediated influx is a key determinant of the intracellular uptake of imatinib but not nilotinib (AMN107): reduced OCT-1 activity is the cause of low in vitro sensitivity to imatinib.. Blood.

[44] White DL, Radich J, Soverini S, Saunders VA, Frede A (2011). Chronic phase chronic myeloid leukemia patients with low OCT-1 activity randomised to high-dose imatinib achieve better responses, and lower failure rates, than those randomized to standard-dose.. Haematologica.

[45] Davies A, Jordanides NE, Giannoudis A, Lucas CM, Hatziieremia S (2009). Nilotinib concentration in cell lines and primary CD34(+) chronic myeloid leukemia cells is not mediated by active uptake or efflux by major drug transporters.. Leukemia.

[46] Giannoudis A, Davies A, Lucas CM, Harris RJ, Pirmohamed M (2008). Effective dasatinib uptake may occur without human organic cation transporter 1 (hOCT1): implications for the treatment of imatinib-resistant chronic myeloid leukemia.. Blood.

[47] Heidel FH, Mack TS, Razumovskaya E, Blum MC, Lipka DB (2012). 3,4-Diarylmaleimides-a novel class of kinase inhibitors-effectively induce apoptosis in FLT3-ITD-dependent cells.. Ann Hematol.

