# Supplementary material for: Intracellular Retention of ABL Kinase Inhibitors Determines Commitment to Apoptosis in CML Cells
Source: PLoS One. 2012 Jul 16;7(7):e40853. doi: 10.1371/journal.pone.0040853 (PMC3397954; doi:10.1371/journal.pone.0040853)
Supplement: Figure S1 — Onset of apoptosis is unchanged upon exposure to high TKI concentrations. Ba/F3-BCR-ABL cells were treated using different TKI concentrations. Cells were lysed at different time-points using lysis buffer and prepared for Western blot analysis. Immunoblotting was performed using monoclonal antibodies specific for total caspase3 and cleaved caspase3. To control for equal loading, blots were stripped and re-probed with anti-actin antibody. (A) depicts results obtained using imatinib (B) depicts results obtained using dasatinib. At least two independent experiments were performed and one representative experiment is shown. (PDF) [file pone.0040853.s001.pdf]

Figure S1

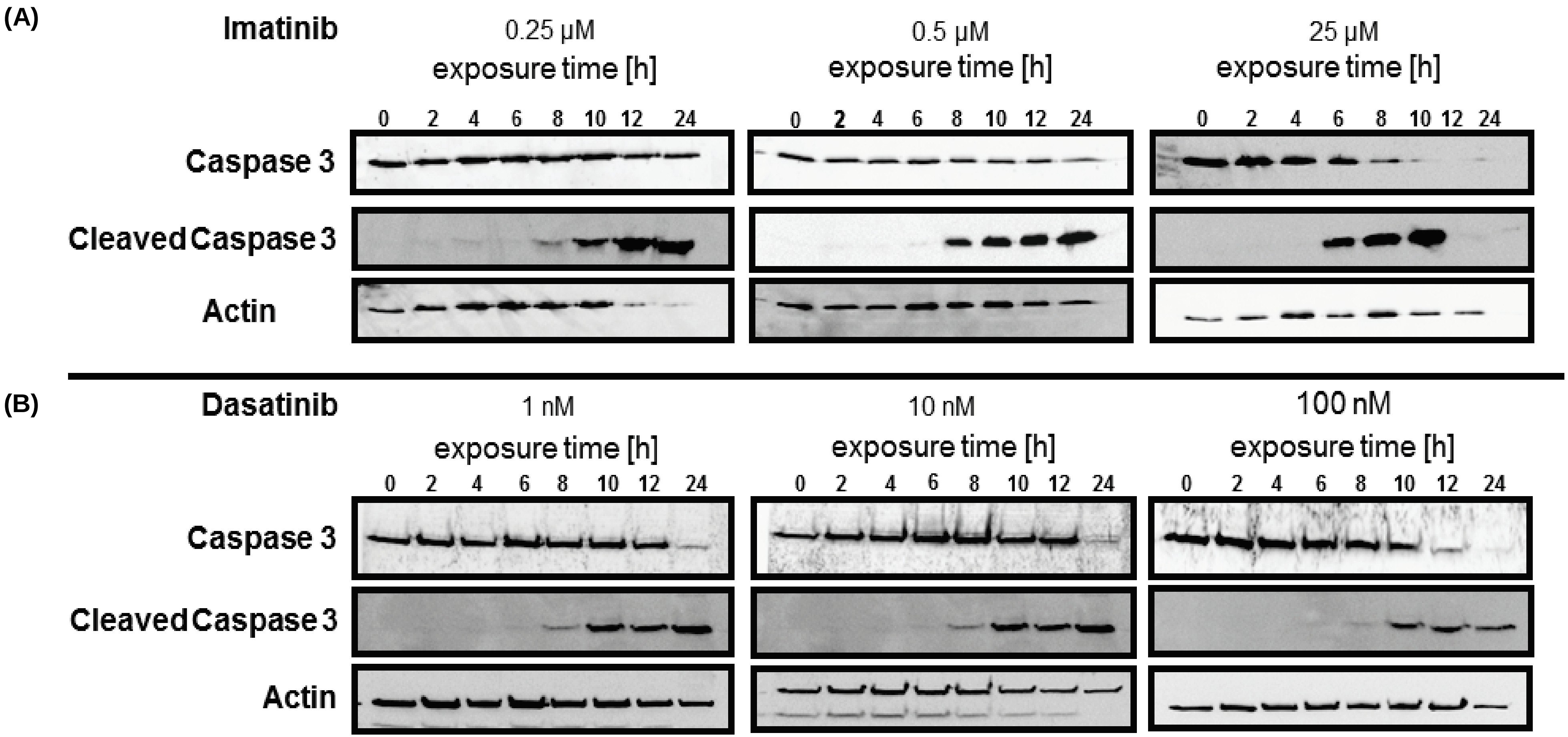
(A)
Imatinib
0.25 µM
exposure time [h]
0.5 µM
exposure time [h]
25 µM
exposure time [h]
0 2 4 6 8 10 12 24
Caspase 3
Cleaved Caspase 3
Actin
(B)
Dasatinib
1 nM
exposure time [h]
10 nM
exposure time [h]
100 nM
exposure time [h]
0 2 4 6 8 10 12 24
Caspase 3
Cleaved Caspase 3
Actin

Figure S1: Onset of apoptosis is unchanged upon exposure to high TKI concentrations

Ba/F3-BCR-ABL cells were treated using different TKI concentrations. Cells were lysed at different time-points using lysis buffer and prepared for Western blot analysis. Immunoblotting was performed using monoclonal antibodies specific for total caspase3 and cleaved caspase3. To control for equal loading, blots were stripped and re-probed with anti-actin antibody.

(**A**) depicts results obtained using imatinib

(**B**) depicts results obtained using dasatinib

At least two independent experiments were performed and one representative experiment is shown.
