# Supplementary material for: Intracellular Retention of ABL Kinase Inhibitors Determines Commitment to Apoptosis in CML Cells
Source: PLoS One. 2012 Jul 16;7(7):e40853. doi: 10.1371/journal.pone.0040853 (PMC3397954; doi:10.1371/journal.pone.0040853)
Supplement: Figure S2 — Control cells do not reveal significant cytotoxic effects upon HD-TKI pulse exposure. (A) Ba/F3-BCR-ABL cells (5×10^4^ cells/ml, total volume 2 ml) were treated with TKI as indicated for 2 h followed by extensive drug wash-out using 2×2 ml PBS. Cells were then re-seeded in 2 ml cell culture medium without TKI. Cells exposed to 0.35% DMSO served as controls (“0 h”). Cells continuously exposed to TKI served as positive controls (“24 h”). Twenty-four hours after start of TKI exposure the percentage of cells in subG1 phase was measured by flow cytometry after propidium iodide staining. Three independent experiments were performed. Data are presented as mean percentage of cells in subG1 phase + SEM. (B) Ba/F3 parental cells (5×10^4^ cells/ml, total volume 2 ml) were treated for 2 h with TKI as indicated followed by thorough drug wash-out using 2×2 ml PBS. Cells were then reseeded in 2 ml cell culture medium without TKI. Twenty-four hours after start of TKI exposure the percentage of cells in subG1 phase was measured by flow cytometry after propidium iodide staining. At least three independent experiments were performed and data are presented as mean percentage of cells in subG1 phase + SEM. (PDF) [file pone.0040853.s002.pdf]

Figure S2

(A)

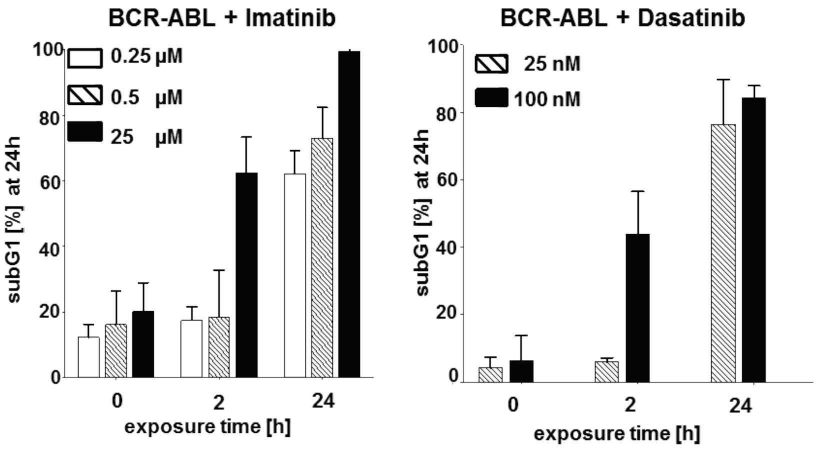

BCR-ABL + Imatinib
0.25 µM
0.5 µM
25 µM
subG1 [%] at 24h
exposure time [h]
BCR-ABL + Dasatinib
25 nM
100 nM
subG1 [%] at 24h
exposure time [h]

(B)

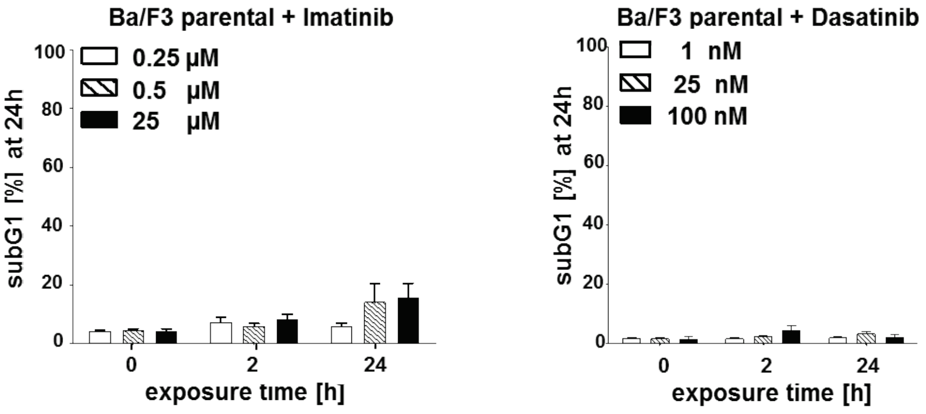

Ba/F3 parental + Imatinib
0.25 µM
0.5 µM
25 µM
subG1 [%] at 24h
exposure time [h]
Ba/F3 parental + Dasatinib
1 nM
25 nM
100 nM
subG1 [%] at 24h
exposure time [h]

Figure S2: Control cells do not reveal significant cytotoxic effects upon HD-TKI pulse exposure

(A) Ba/F3-BCR-ABL cells ($5x10^4$ cells/ml, total volume 2ml) were treated with TKI as indicated for 2h followed by extensive drug wash-out using 2x 2ml PBS. Cells were then re-seeded in 2ml cell culture medium without TKI. Cells exposed to 0.35% DMSO served as controls („0h"). Cells continuously exposed to TKI served as positive controls („24h"). Twenty-four hours after start of TKI exposure the percentage of cells in subG1 phase was measured by flow cytometry after propidium iodide staining. Three independent experiments were performed. Data are presented as mean percentage of cells in subG1 phase +SEM.

(B) Ba/F3 parental cells ($5x10^4$ cells/ml, total volume 2ml) were treated for 2h with TKI as indicated followed by thorough drug wash-out using 2x 2ml PBS. Cells were then reseeded in 2ml cell culture medium without TKI. Twenty-four hours after start of TKI exposure the percentage of cells in subG1 phase was measured by flow cytometry after propidium iodide staining.

At least three independent experiments were performed and data are presented as mean percentage of cells in subG1 phase +SEM.
