# Supplementary material for: Intracellular Retention of ABL Kinase Inhibitors Determines Commitment to Apoptosis in CML Cells
Source: PLoS One. 2012 Jul 16;7(7):e40853. doi: 10.1371/journal.pone.0040853 (PMC3397954; doi:10.1371/journal.pone.0040853)
Supplement: Figure S3 — Repetitive washing prevents apoptosis in K562 cells –effect of a different wash-out protocol. K562 cells were treated either with imatinib or dasatinib as indicated. To control for the effects of different washing protocols, in this case the wash-out procedure was performed as previously described by Shah et al. 2008. In brief, cells (5×10^4^ cells/ml, total volume 2 ml for PI staining and 20 ml for AnnexinV and cleaved caspase3 staining) were washed three times with a volume of medium (containing 10% FCS) that consisted of 50% of the volume of the drug exposure. Cells were afterwards replated in fresh medium (+10% FCS) without inhibitor. For repetitive washing procedures under the same conditions, we generally followed the scheme as is depicted in Figure 1B . (A) Results of PI measurement of cells at 48 hours. Three independent experiments were performed and data are presented as mean percentage of cells in subG1 phase + SEM. (B) FACS measurement of AnnexinV and cleaved caspase3 at 48 hours. The Y-axis represents forward scatter (linear scale) and the X-axis depicts the signal intensity of AnnexinV (left) and cleaved caspase3 (right) on a log-scale. Three independent experiments were performed. One representative experiment is shown. (PDF) [file pone.0040853.s003.pdf]

Figure S3

(A)

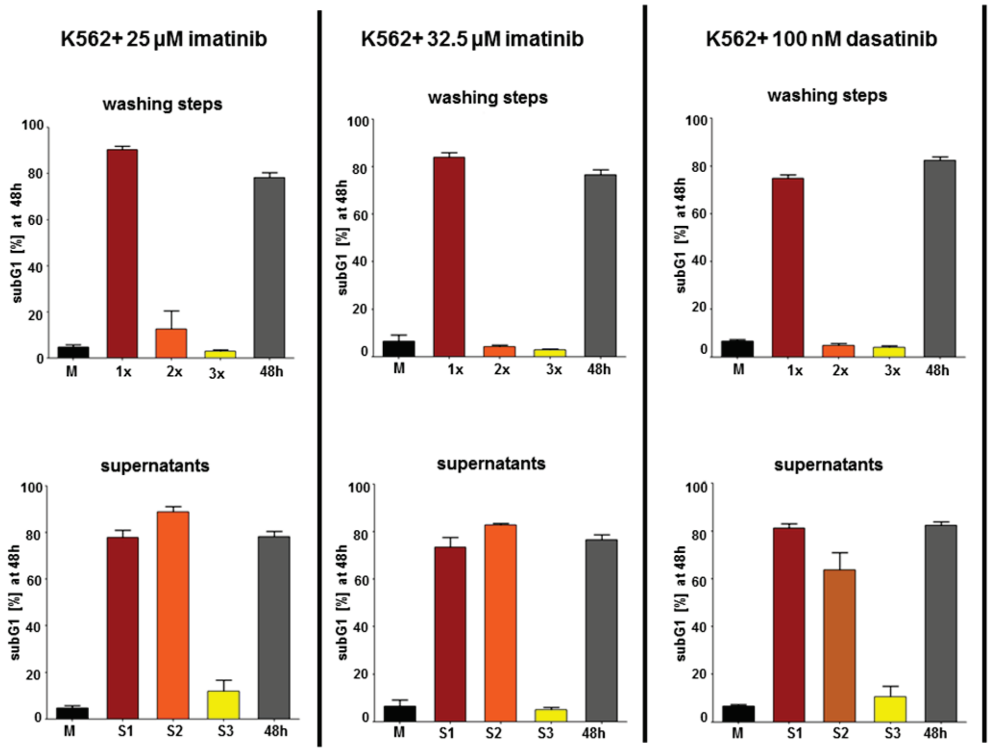

K562+ 25 µM imatinib
K562+ 32.5 µM imatinib
K562+ 100 nM dasatinib
washing steps
supernatants
subG1 [%] at 48h
M
1x
2x
3x
48h
S1
S2
S3

(B)

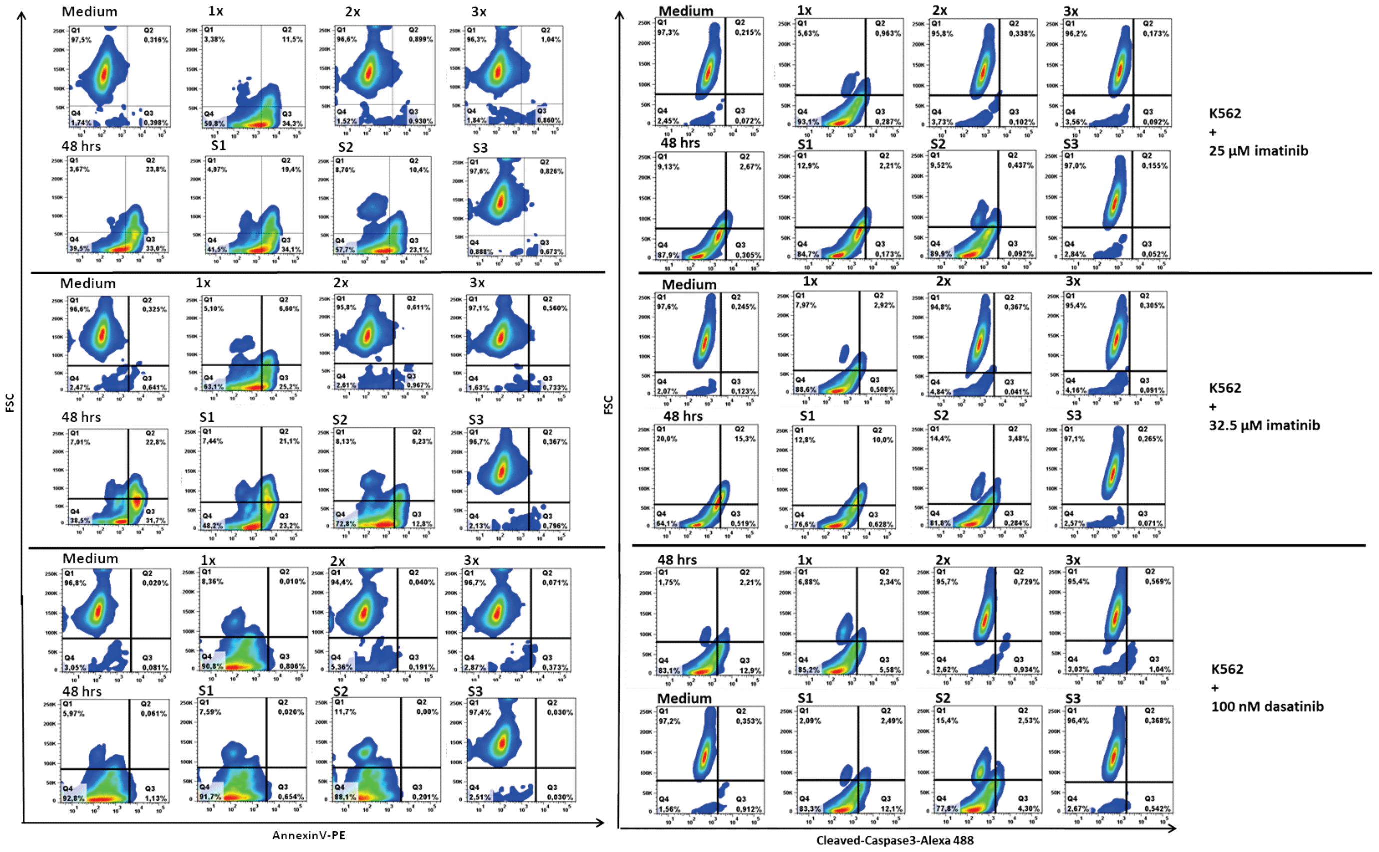

Medium
1x
2x
3x
48 hrs
S1
S2
S3
FSC
AnnexinV-PE
Cleaved-Caspase3-Alexa 488
K562 + 25 µM imatinib
K562 + 32.5 µM imatinib
K562 + 100 nM dasatinib

Figure S3: Repetitive washing prevents apoptosis in K562 cells –effect of a different wash-out protocol

K562 cells were treated either with imatinib or dasatinib as indicated. To control for the effects of different washing protocols, in this case the wash-out procedure was performed as previously described by Shah et al. 2008. In brief, cells ($5x10^4$ cells/ml, total volume 2ml for PI staining and 20 ml for AnnexinV and cleaved caspase3 staining) were washed three times with a volume of medium (containing 10% FCS) that consisted of 50% of the volume of the drug exposure. Cells were afterwards replated in fresh medium (+10% FCS) without inhibitor. For repetitive washing procedures under the same conditions, we generally followed the scheme as is depicted in **Figure 1B.**

(A) Results of PI measurement of cells at 48 hours. Three independent experiments were performed and data are presented as mean percentage of cells in subG1 phase +SEM.

(B) FACS measurement of AnnexinV and cleaved caspase3 at 48 hours. The Y-axis represents forward scatter (linear scale) and the X-axis depicts the signal intensity of AnnexinV (left) and cleaved caspase3 (right) on a log-scale. Three independent experiments were performed. One representative experiment is shown.
