# Supplementary material for: Intracellular Retention of ABL Kinase Inhibitors Determines Commitment to Apoptosis in CML Cells
Source: PLoS One. 2012 Jul 16;7(7):e40853. doi: 10.1371/journal.pone.0040853 (PMC3397954; doi:10.1371/journal.pone.0040853)
Supplement: Figure S4 — Intracellular signaling in K562 cells upon HD-TKI exposure –effect of a different wash-out protocol. K562 cells (5×10^4^ cells/ml, total volume 20 ml) were treated with indicated TKI concentrations. Wash-out was performed as previously described by Shah et al. 2008. In brief, cells were washed three times with medium containing 10% FCS with a volume of medium that consisted of 50% of the volume of the drug exposure. Cells were afterwards replated in fresh medium (+10% FCS) without inhibitor. For repetitive washing procedures under the same conditions we generally followed the scheme as is depicted in Figure 1B . (A) Western Blot analysis of important signaling downstream nodes. Samples were lysed 2 h after each washing step. Untreated cells served as positive controls for phosphorylation signals. Cells treated continuously with TKI for 2 hours or 10 hours (“2 h” and “10 h”) served as positive controls for TKI activity. (B) Cells were treated for 2 h with 100 nM dasatinib, followed by serum wash-out. At various time points after wash-out cells were lysed and prepared for western blot analysis. Phosphotyrosine content was determined using the phosphotyrosine antibodies Y100 and 4G10 as well as P-BCR-ABL (Y177) and (Y412). Antibodies against ABL and GAPDH served as loading control. (PDF) [file pone.0040853.s004.pdf]

Figure S4

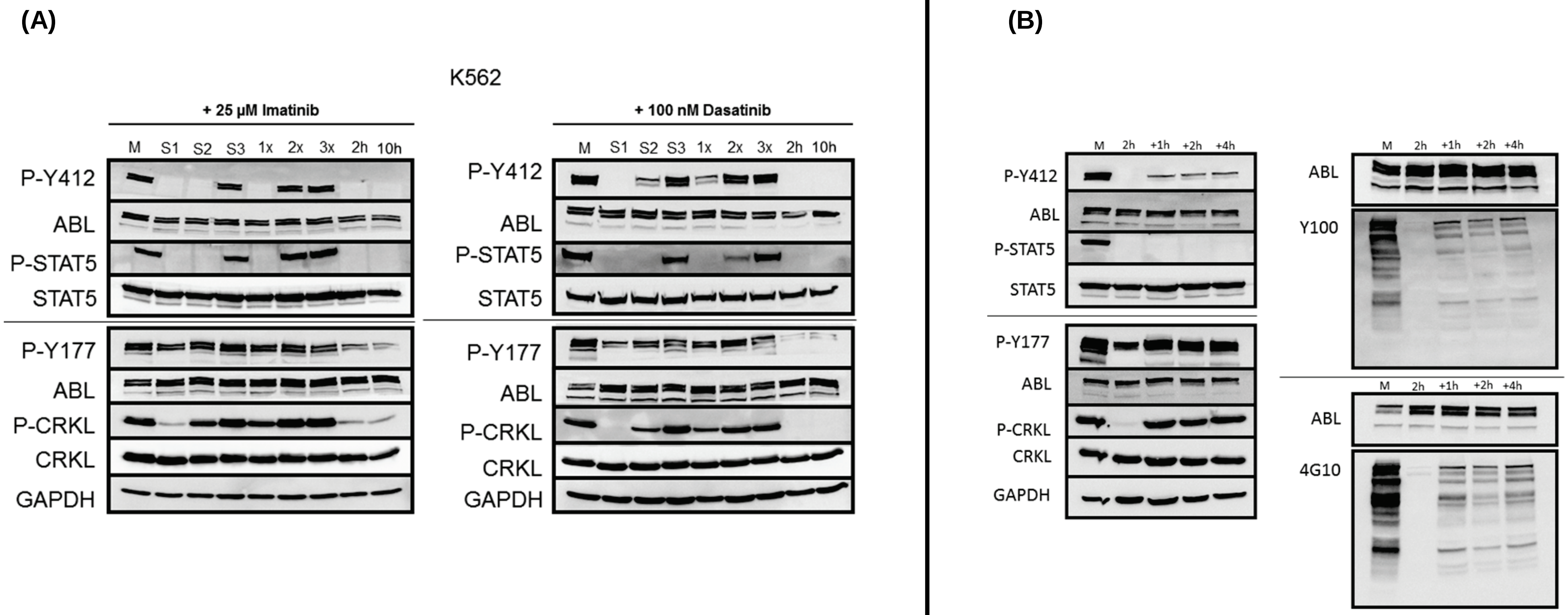

(A)
K562
+ 25 µM Imatinib
+ 100 nM Dasatinib
M S1 S2 S3 1x 2x 3x 2h 10h
P-Y412
ABL
P-STAT5
STAT5
P-Y177
ABL
P-CRKL
CRKL
GAPDH
(B)
M 2h +1h +2h +4h
P-Y412
ABL
P-STAT5
STAT5
P-Y177
ABL
P-CRKL
CRKL
GAPDH
ABL
Y100
ABL
4G10

Figure S4: Intracellular signaling in K562 cells upon HD-TKI exposure –effect of a different wash-out protocol

K562 cells ($5x10^4$ cells/ml, total volume 20ml) were treated with indicated TKI concentrations. Wash-out was performed as previously described by Shah et al. 2008. In brief, cells were washed three times with medium containing 10% FCS with a volume of medium that consisted of 50% of the volume of the drug exposure. Cells were afterwards replated in fresh medium (+10% FCS) without inhibitor. For repetitive washing procedures under the same conditions we generally followed the scheme as is depicted in **Figure 1B**.

(A) Western Blot analysis of important signaling downstream nodes. Samples were lysed 2h after each washing step. Untreated cells served as positive controls for phosphorylation signals. Cells treated continuously with TKI for 2 hours or 10 hours ("2h" and "10h") served as positive controls for TKI activity.

(B) Cells were treated for 2h with 100nM dasatinib, followed by serum wash-out. At various time points after wash-out cells were lysed and prepared for western blot analysis. Phosphotyrosine content was determined using the phosphotyrosine antibodies Y100 and 4G10 as well as P-BCR-ABL (Y177) and (Y412). Antibodies against ABL and GAPDH served as loading control.
