# Supplementary material for: Intracellular Retention of ABL Kinase Inhibitors Determines Commitment to Apoptosis in CML Cells
Source: PLoS One. 2012 Jul 16;7(7):e40853. doi: 10.1371/journal.pone.0040853 (PMC3397954; doi:10.1371/journal.pone.0040853)
Supplement: Figure S5 — Determination of washing efficiency. K562 cells (5×10^4^/ ml) were pulse exposed for 2 h with 25 µM 14C-labeled imatinib followed by wash-out with PBS (1 ml per 5×10^4^ cells per washing step). Immediately after each washing step the PBS supernatant was subjected to beta-counter analysis to measure the concentration of remaining imatinib. After 4 washing steps, cells were replated into TKI free media. Imatinib concentration was then measured 2 h after the last washing step (“+120”). Supernatant analyzed at the end of the TKI exposure (“EOE”) represented a positive control for applied TKI. All measurements were performed in triplicate. Depicted are mean values + SEM of 3 independent experiments. Imatinib concentrations were calculated by fitting the dpm values to a standard curve. (PDF) [file pone.0040853.s005.pdf]

Figure S5

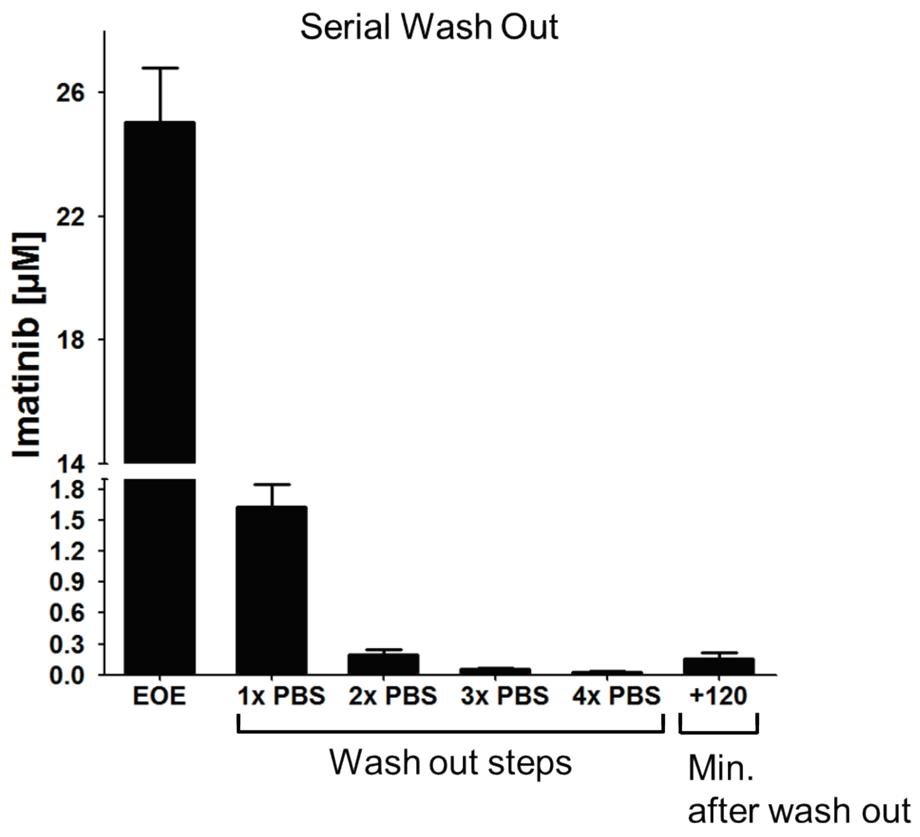

Serial Wash Out
Imatinib [µM]
26
22
18
14
1.8
1.5
1.2
0.9
0.6
0.3
0.0
EOE
1x PBS
2x PBS
3x PBS
4x PBS
+120
Wash out steps
Min.
after wash out

Figure S5: Determination of washing efficiency

K562 cells ($5x10^4$/ml) were pulse exposed for 2h with 25µM ^{14}C-labeled imatinib followed by wash-out with PBS (1ml per $5x10^4$ cells per washing step). Immediately after each washing step the PBS supernatant was subjected to beta-counter analysis to measure the concentration of remaining imatinib. After 4 washing steps, cells were replated into TKI free media. Imatinib concentration was then measured 2h after the last washing step ("+120"). Supernatant analyzed at the end of the TKI exposure ("EOE") represented a positive control for applied TKI. All measurements were performed in triplicate. Depicted are mean values +SEM of 3 independent experiments. Imatinib concentrations were calculated by fitting the dpm values to a standard curve.
