# Supplementary material for: Intracellular Retention of ABL Kinase Inhibitors Determines Commitment to Apoptosis in CML Cells
Source: PLoS One. 2012 Jul 16;7(7):e40853. doi: 10.1371/journal.pone.0040853 (PMC3397954; doi:10.1371/journal.pone.0040853)
Supplement: Figure S6 — ABCB1 expression confers imatinib resistance in K562 cells. K562 and K562-ABCB1 cells were treated continuously either with 0.5 µM or 25 µM imatinib in the presence or absence of 10 µM PSC833 as indicated. Cells exposed to 0.35% DMSO or 10 µM PSC833 alone served as a control. Samples were analyzed after 24 h by flow cytometry after propidium iodide staining. Three independent experiments were performed and results are shown as means ± SEM. (PDF) [file pone.0040853.s006.pdf]

Figure S6

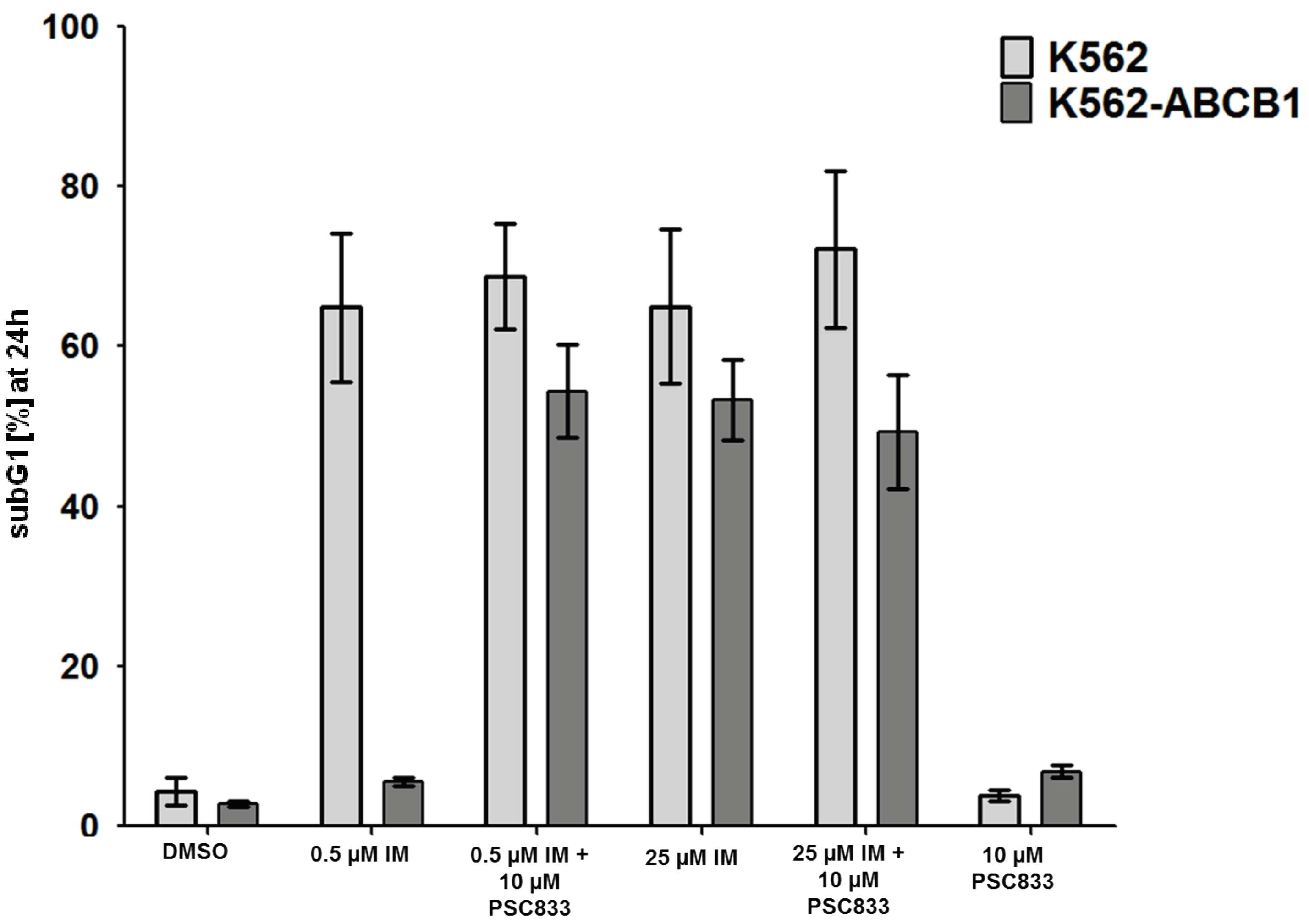

100
80
60
40
20
0
subG1 [%] at 24h
K562
K562-ABCB1
DMSO
0.5 µM IM
0.5 µM IM + 10 µM PSC833
25 µM IM
25 µM IM + 10 µM PSC833
10 µM PSC833

Figure S6: ABCB1 expression confers imatinib resistance in K562 cells

K562 and K562-ABCB1 cells were treated continuously either with 0.5µM or 25µM imatinib in the presence or absence of 10µM PSC833 as indicated. Cells exposed to 0.35% DMSO or 10µM PSC833 alone served as a control. Samples were analyzed after 24h by flow cytometry after propidium iodide staining. Three independent experiments were performed and results are shown as means ± SEM.
