# Supplementary material for: Intracellular Retention of ABL Kinase Inhibitors Determines Commitment to Apoptosis in CML Cells
Source: PLoS One. 2012 Jul 16;7(7):e40853. doi: 10.1371/journal.pone.0040853 (PMC3397954; doi:10.1371/journal.pone.0040853)
Supplement: Table S1 — TKI concentrations measured by HPLC/LC-MS/MS in cellular lysates. (A) TKI concentrations ± SEM measured by HPLC/LC-MS/MS in cellular lysates treated with 25 µM imatinib or 100 nM dasatinib for 2 h as indicated followed by drug wash-out. Intracellular TKI concentrations were calculated based on the mean cellular volume. (B) Mean size in µm ± SD of Ba/F3-BCR-ABL cells and K562 cells. In total, 100 cells of each cell line were measured. Intracellular imatinib concentration was calculated based on the assumption that cells are spherical (V = 4/3×π×r3). Thus, the total cellular volume for 0.2×10^6^ cells is 0.19 µl for Ba/F3-BCR-ABL cells and 0.5 µl for K562 cells. (PDF) [file pone.0040853.s007.pdf]

Table S1

(A)

	$C_{imatinib}$ [µM] Ba/F3-BCR-ABL	$C_{imatinib}$ [µM] K562	$C_{dasatinib}$ [µM] K562
Lysate (diluted)	0.52 (±0.09)	0.81 (±0.23)	0.000583 (±0.00004)
Intracellular concentration (calculated)	10994	6513	4.688

(B)

	Diameter [µM]	Calculated volume/cell [L]
Ba/F3-BCR-ABL	12.18 (±1.63)	0.946×10^{-12}
K562	16.81 (±1.93)	2.487×10^{-12}

Table S1: TKI concentrations measured by HPLC / LC-MS/MS in cellular lysates

(A) TKI concentrations ±SEM measured by HPLC / LC-MS/MS in cellular lysates treated with 25µM imatinib or 100nM dasatinib for 2h as indicated followed by drug wash-out. Intracellular TKI concentrations were calculated based on the mean cellular volume.
(B) Mean size in µm ±SD of Ba/F3-BCR-ABL cells and K562 cells. In total, 100 cells of each cell line were measured. Intracellular imatinib concentration was calculated based on the assumption that cells are spherical ($V = 4/3 \times \pi \times r^3$). Thus, the total cellular volume for 0.2×10^6 cells is 0.19µl for Ba/F3-BCR-ABL cells and 0.5µl for K562 cells.
